# Spatially resolved transcriptomics in human brain metastases identifies macrophage-tumor interactions associated with survival

**DOI:** 10.1016/j.isci.2026.116517

**Published:** 2026-08-12

**Authors:** Aaditya Khatri, Courtney M. McKernan, Amanda E.D. Van Swearingen, Vaibhav Jain, Hannah L. Thrash, Arianna Towne, Jing Jin Gu, Simon G. Gregory, Carey K. Anders, Ann Marie Pendergast

**Affiliations:** 1 Department of Medicine, Division of Pulmonary, Allergy and Critical Care Medicine, Duke University School of Medicine, Durham, NC, USA; 2 Department of Pharmacology and Cancer Biology, Duke University School of Medicine, Durham, NC, USA; 3 Duke Center of Brain and Spine Metastasis, Duke Cancer Institute, Durham, NC, USA; 4 Duke Molecular Physiology Institute, Duke University, Durham, NC, USA; 5 Department of Neurosurgery, Duke University School of Medicine, Durham, NC, USA

**Keywords:** brain metastasis, tumor microenvironment, spatial transcriptomics

## Abstract

Despite advances in treatment approaches, the mean survival for patients with brain metastases remains poor. The incidence of brain metastases continues to rise, and there remains a need to identify novel therapeutics targeting mechanisms critical for brain metastasis. We employed a multi-omic approach, including single nuclei and spatially resolved transcriptomic profiling across 23 brain metastatic samples, with the representation of lung, breast, and melanoma metastases, to identify tumor-microenvironment interactions associated with survival outcomes in brain metastasis. We found that the specific role of macrophages in disease progression is context-dependent. Activated HLA-DR+ inflammatory macrophages directly in contact with cancer cells at the tumor boundary are associated with responsiveness to therapies and improved patient survival. Conversely, reprogrammed macrophages expressing extracellular matrix proteins and TGFβ1 are associated with poor survival. These findings identify spatially distinct tumor cell-macrophage interactions associated with survival outcomes in patients with brain metastases and represent targets for immunotherapy strategies.

## Highlights

• Multi-omic analysis identifies spatially distinct macrophages in human brain metastases
• Patients with BrM with good vs. poor survival have distinct macrophage transcriptional profiles
• Inflammatory macrophages at the tumor boundary are associated with improved patient survival
• Macrophages expressing ECM proteins and TGFβ1 are associated with poor survival

## Graphical abstract

**Figure:**
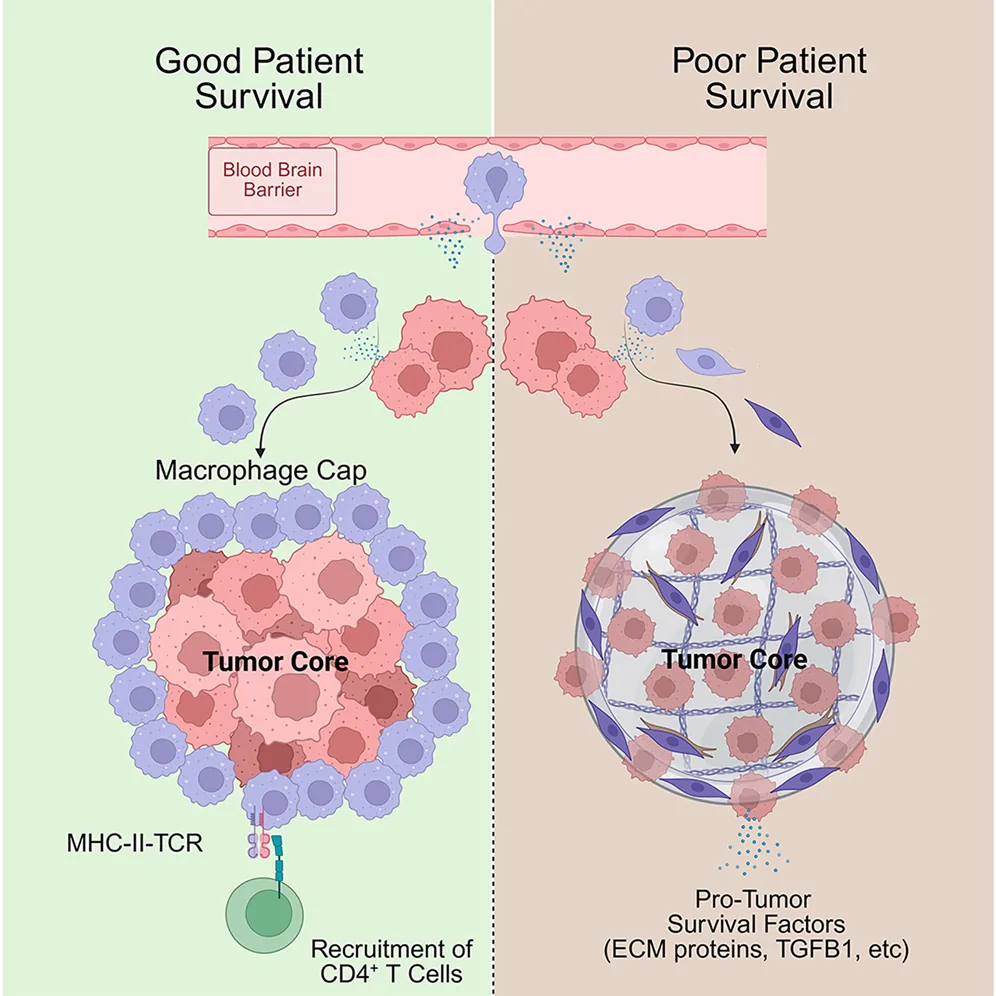

## Teaser

Microenvironment; Neuroscience; Omics

## Introduction

Brain metastases account for the majority of intracranial tumors with an estimated 170,000 to 200,000 cases diagnosed each year in the United States.^1^^,^^2^^,^^3^^,^^4^^,^^5^^,^^6^ Despite advances in treatment approaches and improved survival for patients with cancer over the last few decades, the mean survival for patients with brain metastases remains 6–8 months even in the most recent studies.^7^^,^^8^ The incidence of brain metastases has increased, potentially due to increased overall patient survival and poor blood-brain barrier penetrance of systemic therapies.^9^^,^^10^ Older autopsy studies showed evidence of brain metastases in as many as 40% of patients with cancer at the time of death, though the true modern rate may be higher.^11^ Therefore, there remains a critical unmet need to identify novel therapeutics targeting tumor intrinsic pathways and cell-cell interactions driving brain metastases.

Advances in sequencing technologies, including high-dimensional, single-cell analyses of brain metastases, have dramatically enhanced our understanding of both tumor and micro-environment cell types in human brain metastases.^12^^,^^13^^,^^14^^,^^15^ However, there remains a need to integrate these large datasets with important histopathologic and clinical characteristics to better understand the cell-cell interactions in spatial context that drive disease progression. For example, isolation and processing of cancer cells from a tumor core with >90% malignant cells may yield a different transcriptomic and proteomic landscape than isolation and processing of cancer cells from the tumor boundary, where specific cell-cell interactions may uncover the mechanisms by which the tumor is able to invade the brain parenchyma.

Here, we used a multi-omic approach, including single-nuclei sequencing and spatial transcriptomic analysis with immunofluorescence validation to identify cell-cell interactions at the leading edge of the tumor, where tumor cells directly interface with the surrounding brain microenvironment. We identified HLA-DR-high, pro-inflammatory, monocyte-derived macrophages (MDMs) at the tumor boundary that correlate with improved patient survival across human lung and breast cancer brain metastases. Further, we found morphologically and functionally distinct pro-tumorigenic macrophages within the tumor associated with poor survival among patients with brain metastases. These findings identify spatially distinct tumor cell-macrophage interactions associated with survival in patients with brain metastases, which could represent potential targets for immunotherapy strategies.

## Results

### A repository of brain metastatic samples for multi-omic analysis

**Figure 1:**
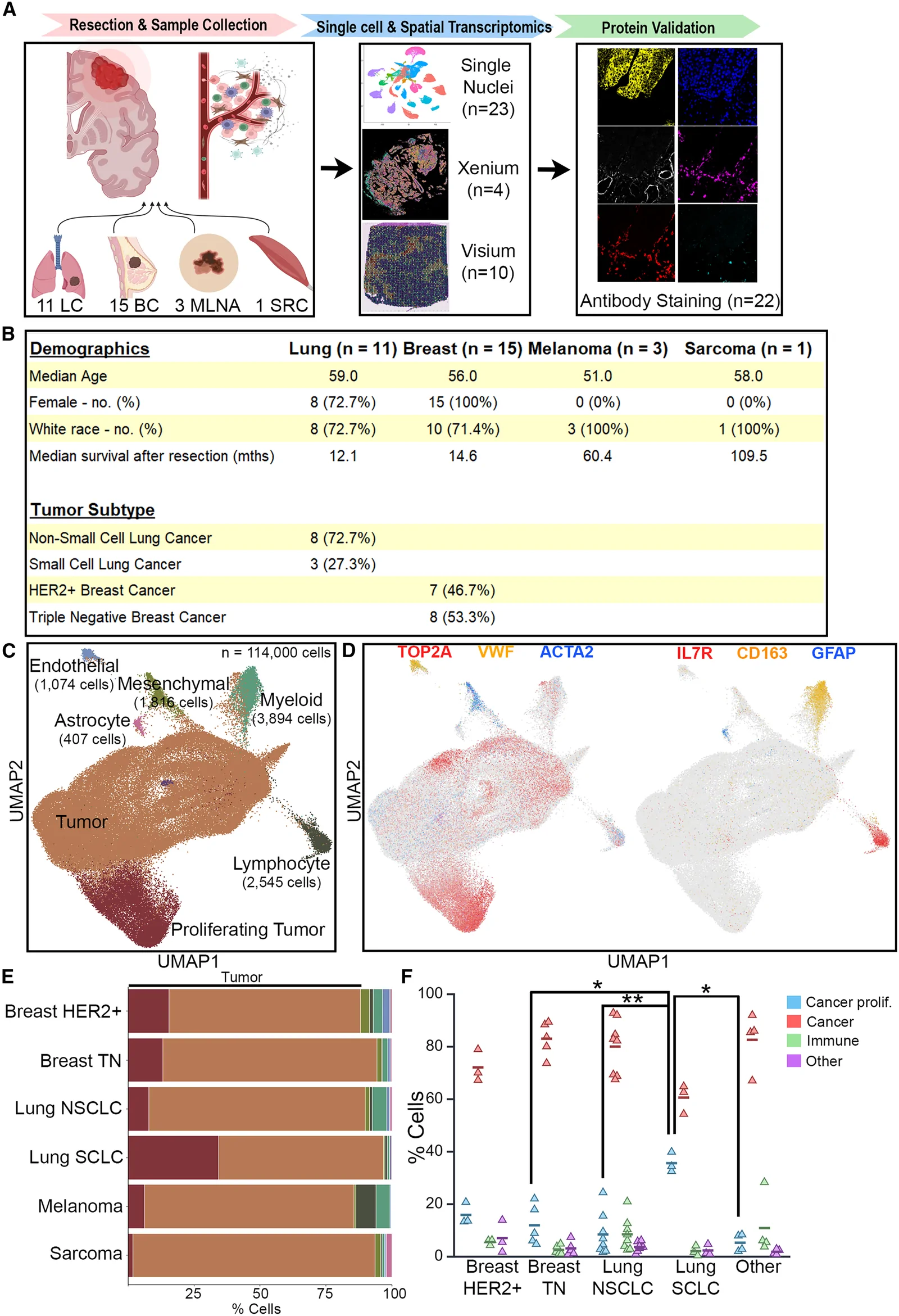
Overview of brain cancer metastasis dataset (30 samples) (A) Schematic of the experimental design, created with Biorender.com. Samples included lung cancer (LC), breast cancer (BC), melanoma (MLNA), and sarcoma (SRC) brain metastases. (B) Clinical characteristics of 30 patients with brain metastatic resection. (C and D) UMAP visualization of cell types of 23 samples submitted for single nuclei sequencing. (E) Distribution of cell types by cancer type. (F) Two-way ANOVA followed by post-hoc Tukey testing shows significant enrichment of proliferating cancer cells in small cell lung cancer samples. *p* value <0.05 (∗) and *p* value <0.01 (∗∗).

We identified 30 human brain metastasis specimens resected at Duke University Medical Center from 1998 to 2022 for single-nuclei and spatial transcriptomic analysis as well as protein validation studies (Figure 1A). Specimens were selected following histopathologic and clinical review (Table S1). The median age at the time of resection was 57 years, while the median survival after resection was 14 months. Four patients had exposure to chemotherapy or radiation within one month prior to resection. Among the specimens, 23 brain metastasis specimens passed quality control for single nuclei sequencing analysis, encompassing eleven lung cancer (eight non-small cell lung cancer [NSCLC], three small cell lung cancer [SCLC]), eight breast cancer (three HER2+, five triple negative [TNBC]), three melanoma, and one sarcoma brain metastasis (Figure 1). A broad graph-based uniform manifold approximation and projection (UMAP) of 114,000 cells from all 23 brain metastasis specimens identified tumor cells (∼80% of all cells), subdivided into proliferating (enriched in *TOP2A*) and non-proliferating clusters as well as cell types in the tumor microenvironment (TME), including myeloid, lymphocyte, astrocyte, endothelial, and mesenchymal cells (Figures 1C–1F). Total cell counts are shown in Table S2 and Figure S1A. Within tumor sub-types, the only significant change in the proportion of cell types within these clusters was an increase in proliferating tumor cells in the SCLC brain metastases (Figures 1E and 1F).

### Transcriptional landscape of tumor cells in brain metastatic disease

**Figure 2:**
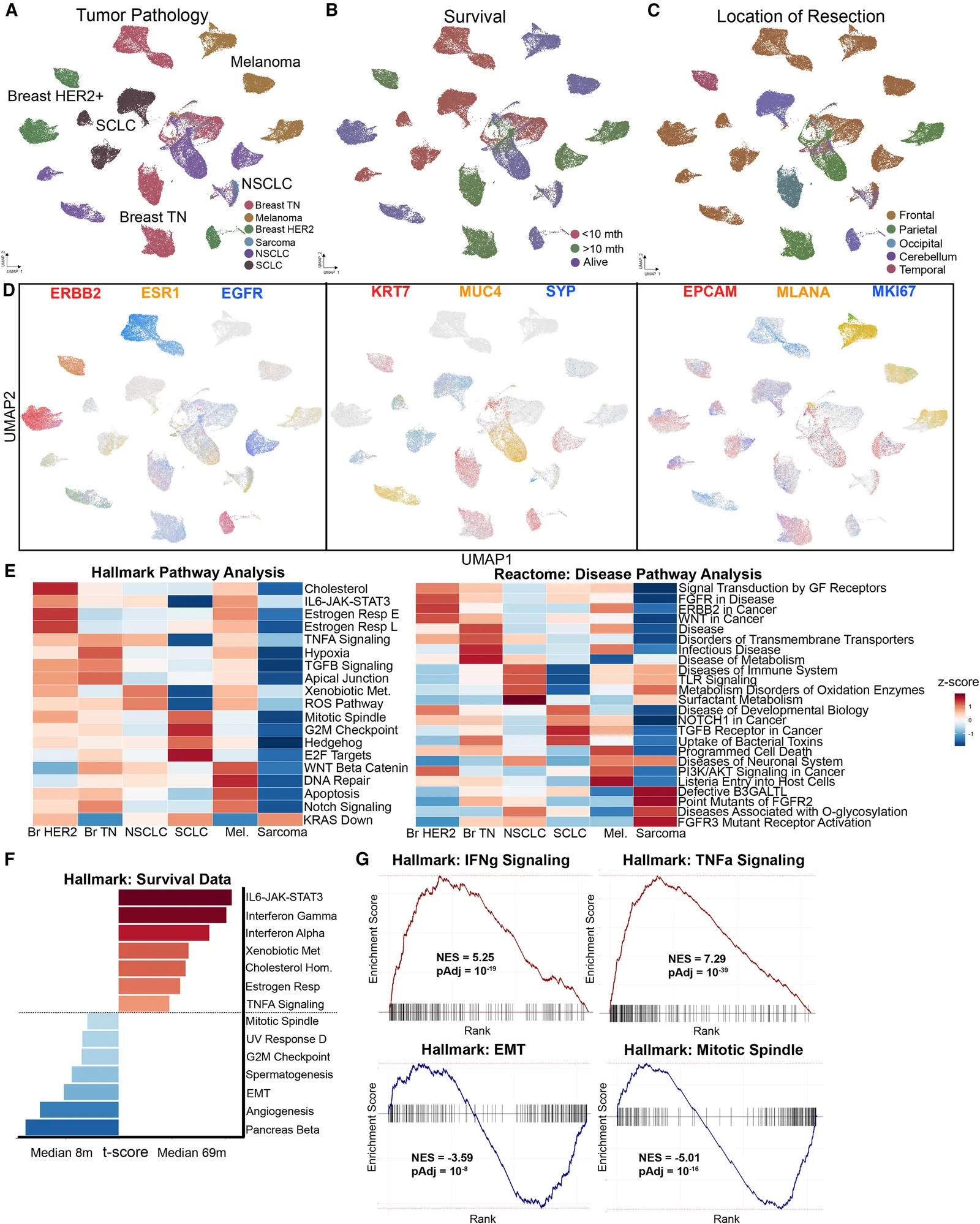
Characterization of tumor cell types (A–C) UMAP projections based on tumor types, survival, and location of tumor resection. (D) Expression of genes characterizes cell clusters, including breast cancer markers (*ERBB2*, *ESR1*), lung cancer markers (*MUC4*), and melanoma markers (*MLANA*). (E) Hallmark and Reactome pathway analyses identify specific pathways enriched in each tumor cell type. (F and G) Hallmark pathway analysis and GSEA show the upregulation of inflammatory pathways (Interferon, JAK-STAT, TNF alpha signaling) in patients with good survival (>24 months (*n* = 9)) and upregulation of pro-tumorigenic pathways (angiogenesis, EMT, and mitosis) in patients with poor prognosis (<24 months (*n* = 10)).

We next sought to characterize the transcriptional landscape of the tumor cells across all brain metastases specimens. We performed sub-cluster analysis followed by pathway analysis of the tumor cell types, which showed expected upregulation of genes and pathways in tumor sub-types (Figures 2A–2E and S1). HER2+ BC samples demonstrated the upregulation of *ERBB2* (Figure 2D) and ERBB2 and estrogen response signaling (Figures 2E and S1), while SCLC samples demonstrated the upregulation of the neuroendocrine marker, *SYP* (Figure 2D) and cell proliferation pathways promoting cell division (Figures 2E and S1). To assess whether specific transcriptional patterns were associated with survival across cancer types, we performed pathway analysis comparing tumor cells from patients with good survival (>24 months, median 69 months) to those with poor survival (<24 months, median 8 months). We found the upregulation of inflammatory pathways, including interferon, TNF, and JAK-STAT signaling, was associated with good survival outcomes, while the upregulation of angiogenesis, EMT, and cell cycle pathways was associated with poor survival outcomes (Figures 2B, 2F, 2G, and S2). Previous studies have found that brain metastatic cells can exhibit neural characteristics to generate favorable neural microenvironments upon the colonization of the brain parenchyma.^16^ Consistent with these findings, the upregulation of GPCR-glutamate signaling, nerve growth signaling, and netrin activation correlated with poor survival (Figure S2B).

### Transcriptional landscape of microenvironment cell types in brain metastatic disease

**Figure 3:**
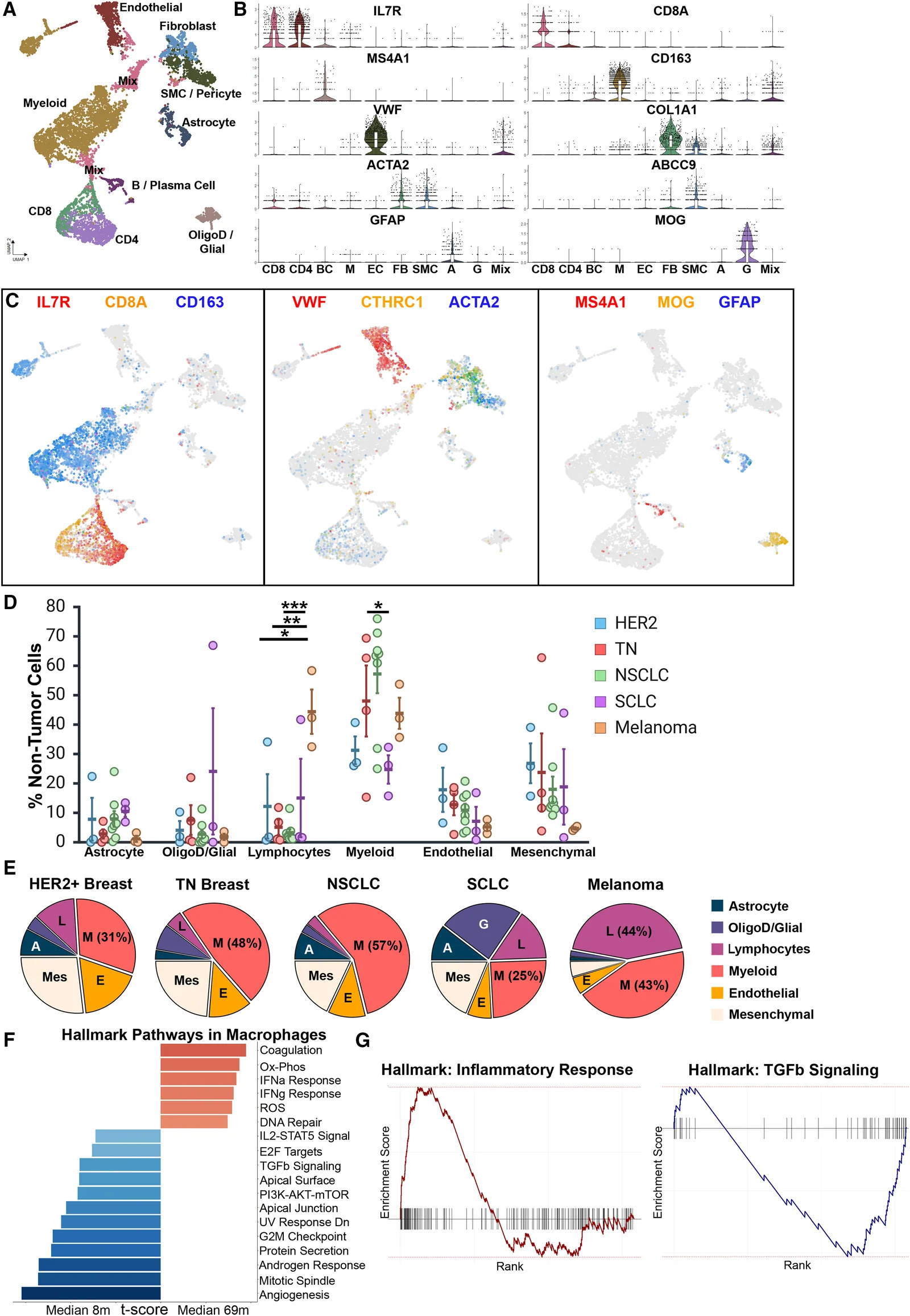
Characterization of cell types in the tumor microenvironment of brain metastases (A) UMAP projection of microenvironment cell types in 23 brain metastases samples. (B and C) Violin and feature plots show gene expression of genes characterizing each cell type: *IL7R* (CD4 T cells)*, CD8A* (CD8 T cells)*, CD163* (macrophages)*, VWF* (endothelium)*, CTHRC1* (fibroblasts)*, ACTA2* (smooth muscle cells)*, MS4A1* (B cells)*, MOG* (oligodendrocytes)*,* and *GFAP* (astrocytes and glial cells). (D) Two-way ANOVA followed by post-hoc Tukey testing shows significant enrichment of lymphocytes in melanoma brain metastases and myeloid cells in NSCLC compared to SCLC metastases. *p* value <0.05 (∗), *p* value <0.01 (∗∗), and *p* value <0.001 (∗∗∗). (E) Pie charts show the distribution of microenvironment cell types in each cancer. (F and G) Survival data pathway analysis shows the upregulation of inflammatory pathways (IFN signaling) in patients with good prognosis and upregulation of pro-tumorigenic pathways (angiogenesis, mitosis, TGFβ signaling) in patients with poor prognosis.

We next characterized the TME cell types in the single nuclei datasets, and found the representation of immune (CD4 T cells, CD8 T cells, B cells, astrocytes, oligodendrocytes, myeloid cells), mesenchymal (smooth muscle cells, fibroblasts, pericytes), and endothelial cells (Figures 3A–3C and S3). Comparison of the proportion of TME cells by tumor type showed a significant increase in the representation of lymphocytes in the three melanoma brain metastasis specimens versus other tumor types (Figures 3D and 3E). Notably, the three patients with melanoma with brain metastases exhibited improved survival (median 60 months) compared to all other patients with brain metastases (median 14 months). Single-nuclei RNA-seq analysis of the distribution of cell types in the TME by patient survival did not show any statistically significant changes associated with survival outcomes (Figure S3E).

### Reprogramming of macrophages is associated with poor survival across brain metastases

As myeloid cells represented the largest proportion of immune cells in the single nuclei RNA-seq dataset, we performed further analyses in this cell type. Post-hoc testing found a significant increase in myeloid cells in NSCLC compared to SCLC brain metastases specimens, but did not show significant differences in the proportion of other cell types across cancer sub-types (Figures 3D and 3E). While known to constitute a major myeloid population of the normal human brain, microglia (as denoted by the expression of *P2RY12* or *SALL1*) only comprised ∼4% of the myeloid population (Figure S3B).^17^ Notably, pathway analysis comparing patients with good survival (median 69 months) and poor survival (median 8 months) showed profound changes in the transcriptional profiling of macrophages. We found a significant increase in pro-inflammatory pathways in macrophages associated with good survival outcomes, while an increase in pro-tumorigenic pathways, including angiogenesis, regulation of mitotic spindle, and TGFβ signaling in macrophages associated with poor survival outcomes (Figures 3F and 3G).

### Spatially resolved transcriptomics identifies the enrichment of macrophages at the leading edge of brain metastases

**Figure 4:**
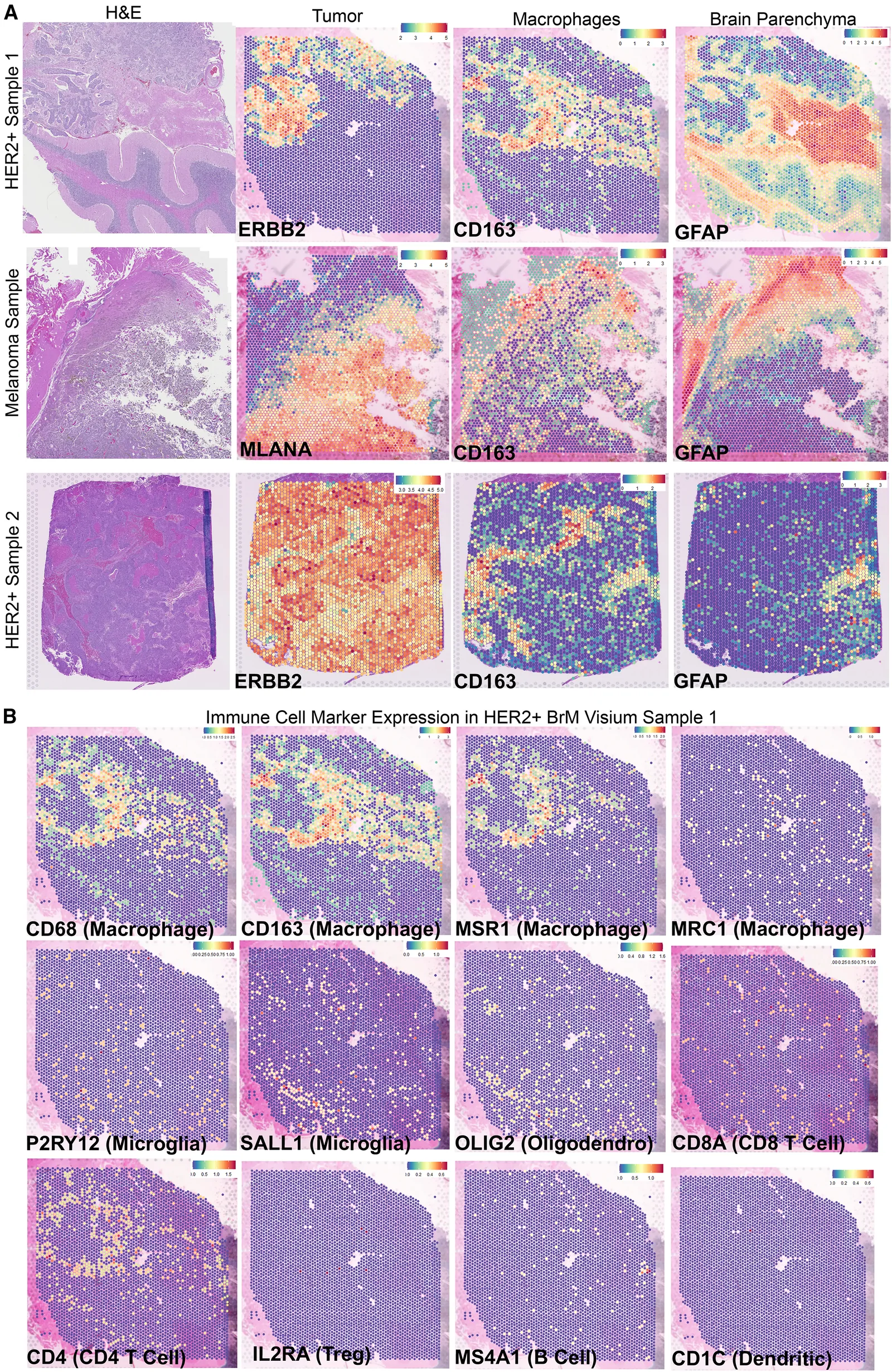
Visium spatial transcriptomic analysis in brain metastatic samples identifies the enrichment of macrophages along the tumor boundary (A) H&E and feature plot show the distribution of cancer cells (*ERBB2* or *MLANA*), macrophages (*CD163*), and neurons (*GFAP*) across 3 Visium spatial transcriptomic samples selected for the presence of tumor-brain parenchyma interface. (B) Feature plot shows distribution of immune cell markers in the HER2+ BrM sample 1 shown in Figure 4A (top panels). In addition to the enrichment of macrophages, there is enrichment of CD4 T cells.

To better characterize the macrophage populations in brain metastases, we performed spatially resolved transcriptomics using the 10X Genomics Visium (*n* = 12) and Xenium (*n* = 4) platforms on a subset of the brain metastasis specimens. In total, we performed Visium spatial transcriptomic analysis on twelve samples (ten independent patient samples, with two specimens run in replicate from the same patients) (Table S1; Figure S4). Seven brain metastasis specimens were taken from the tumor core, which contained >90% tumor cells, without any evidence of the tumor margin, while five specimens included the tumor margin with a significant proportion of brain parenchyma (Figure S4). To better understand the spatial distribution of myeloid cells and other immune cell populations, we analyzed gene expression markers of macrophages (*CD163*, *CD68*, *MSR1*, *MRC1*), microglia (*P2RY12*, *SALL1*), oligodendrocytes (*OLIG2*), and lymphocytes (*CD8A*, *CD4*, *IL2RA*). We were particularly interested in genes enriched at the interface of the tumor (*ERBB2*, *MLANA*) and brain parenchyma (*GFAP*) (Figures 4 and S5). We found a profound enrichment of a subset of macrophage markers, *CD68*, *CD163*, and *MSR1*, at the leading edge of the tumor and within the margins of large vessels (Figure 4A). These cells do not express microglia markers, including *P2RY12* and *SALL1* (Figures 4B and S5), which have been used to identify resident microglia populations in previous single-cell brain metastasis studies.^13^^,^^18^ Thus, the majority of myeloid cells in our single nuclei and spatial datasets likely represent MDMs crossing the blood-brain barrier from circulation into the brain parenchyma. We found no enrichment of oligodendrocytes, cytotoxic CD8^+^ T cells, T regulatory cells, B cells, or dendritic cells at the tumor boundary. Consistent with the enrichment of antigen-presenting cells, we found an enrichment of CD4^+^ T cells in a subset of brain metastases (Figures 4B and S5).

**Figure 5:**
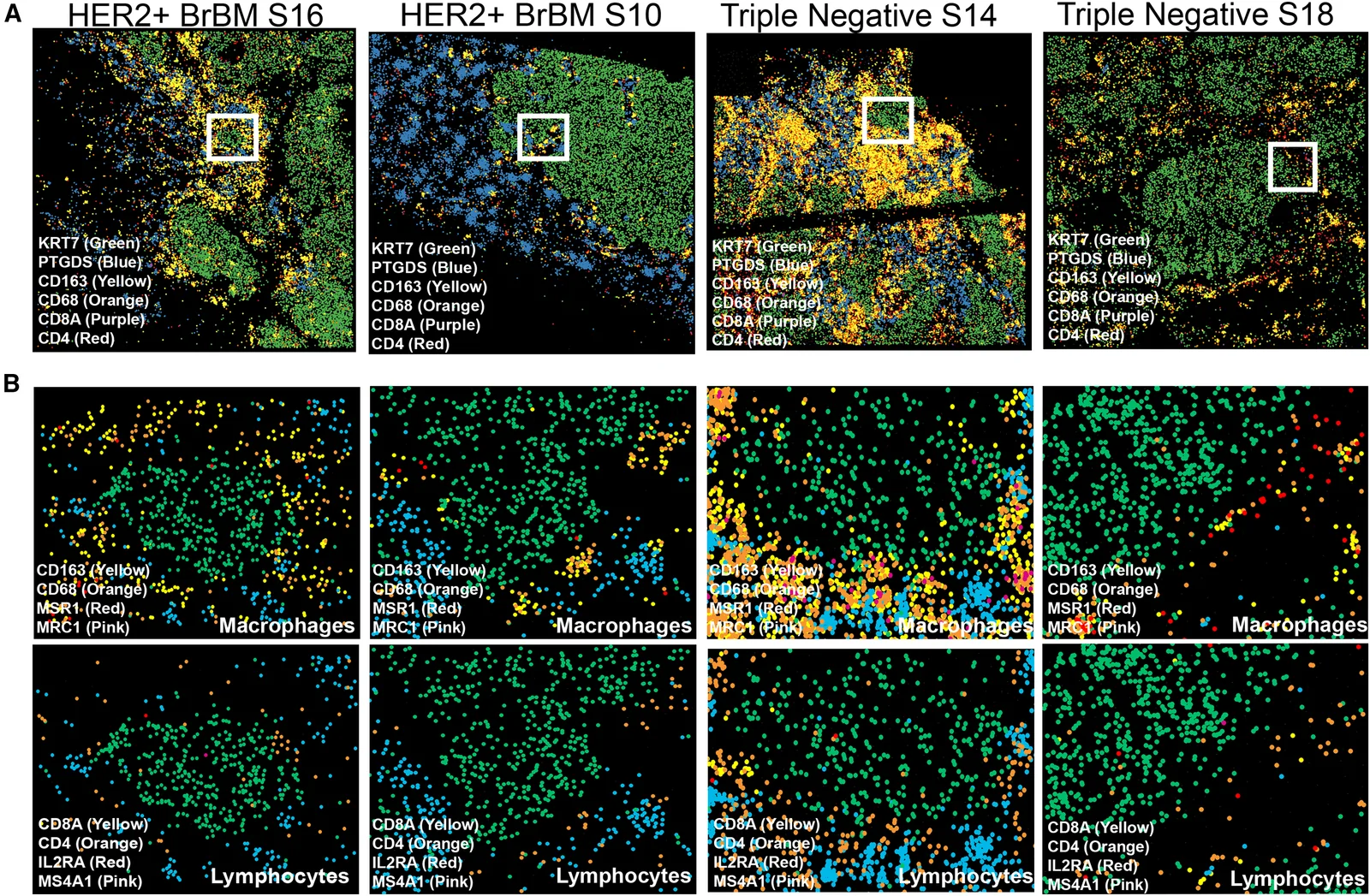
Xenium spatial transcriptomic analysis identifies the enrichment of macrophages at the tumor boundary (A and B) Feature plot shows the enrichment of a subset of macrophage markers (*CD68*, *CD163*, *MSR1*) compared to lymphocyte markers at the leading edge of the tumor (*KRT7,* green) in Xenium spatial transcriptomic samples. Brain parenchyma is indicated in blue by the expression of *PTGDS*. The white box in (A) is the zoomed in region shown in (B).

To assess whether distinct cell-cell interactions between macrophages and cancer cells are found at the leading edge of the brain metastatic tumors, we next performed spatially resolved transcriptomic analysis at single-cell resolution on the 10X Xenium platform. Though sequencing on the Visium platform provided transcriptomic data across 20,000 genes, a limitation of this platform is that it provides spatial resolution only to spots 50 μm in size. In contrast, the Xenium spatial transcriptomic technology allows for targeted gene expression analysis with single-cell spatial resolution. We performed sequencing of two HER2+ BC brain metastases and two TNBC brain metastases specimens on the Xenium platform. Three of the four samples were distinct samples not used in the prior Visium or single nuclei transcriptomic analyses. For the analysis, we curated a panel of 280 genes targeted for analysis in breast cancer samples (Table S3). These include breast cancer cell markers (*ERBB2*, *ESR1*, *PGR*, *EGFR*, *MKI67*, *TOP2A*), immune cell markers (*PTPRC*, *CD163*, *CD68*, *CD4*, *CD8A*), endothelial cell markers (*PECAM1*, *VWF*), and mesenchymal markers (*MYH11*, *ACTA2*, *POSTN*, *LUM*), among others. We identified *PTGDS*, encoding prostaglandin D2 synthase, as the gene most enriched in the brain parenchyma and used this marker to identify tumor margins (Figures S6 and 5A). Consistent with Visium spatial transcriptomic results, macrophage-associated transcripts *CD163*, *CD68*, and *MSR1* were enriched at the tumor boundary (*KRT7*) (Figure 5B), indicating a recruitment of macrophages to the tumor leading edge.

### Macrophage recruitment to the tumor boundary is associated with improved survival

**Figure 6:**
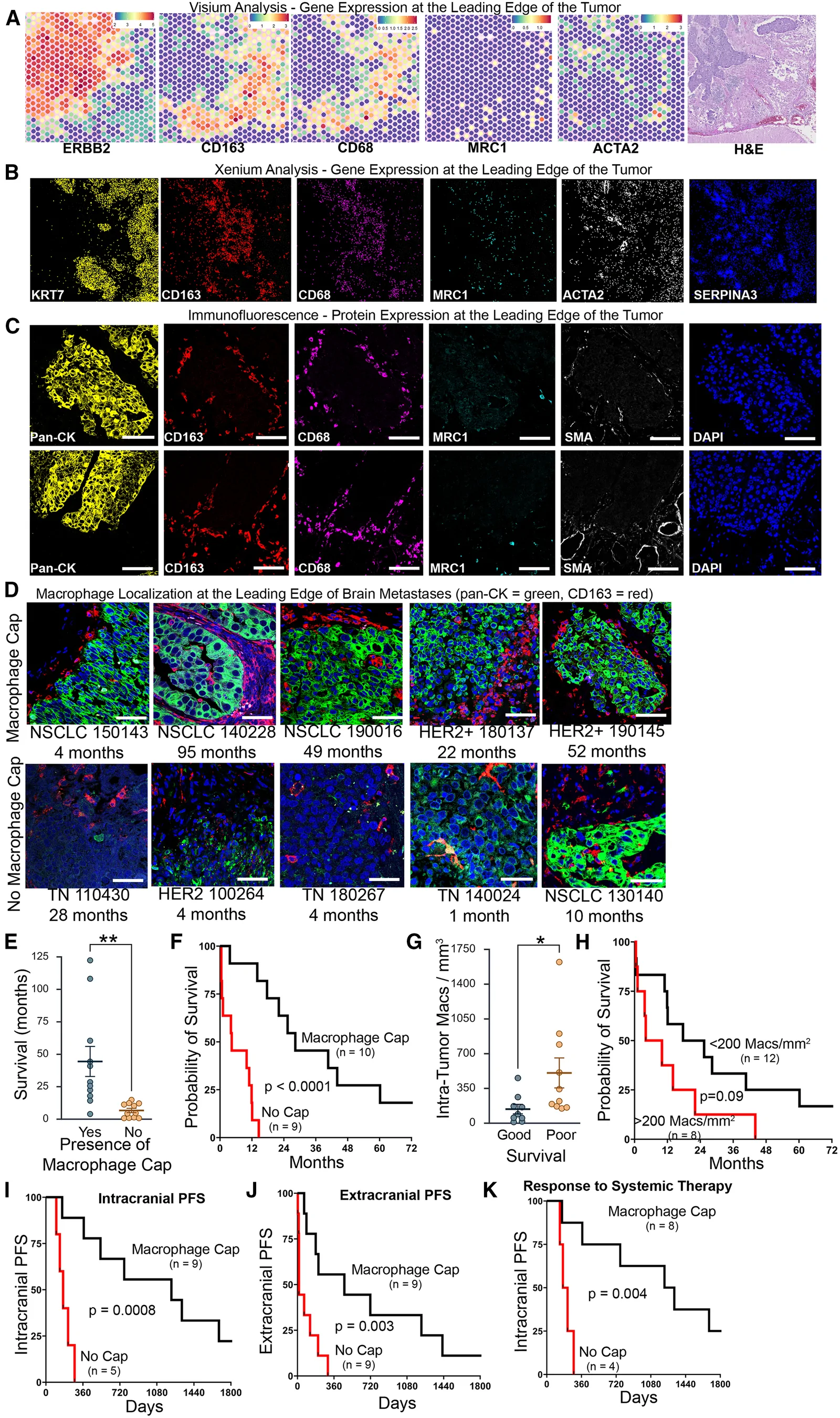
Immunofluorescence validation of macrophage subsets in brain metastatic samples (A and B) Visium (A) and Xenium (B) spatial transcriptomic analysis shows the enrichment of a subset of macrophage markers (*CD68*, *CD163,* but not *MRC1*) at the tumor boundary. (C) Sequential staining of a HER2+ brain metastatic sample confirms the enrichment of a sub-population of macrophages (CD163^+^, CD68^+^, MRC1-) at the leading edge of the tumor (pan-CK, yellow) of two separate regions from the same sample. (D–H) Enrichment of macrophages (CD163, red) at the tumor leading edge (pan-CK, green)—a “macrophage cap” (E–F)—is associated with improved survival across brain metastatic samples. Log rank (Mantel-Cox) testing showed a statistically significant difference in survival between tumors with macrophage caps and those without. However, the enrichment of macrophages within the tumor is associated with poor survival across brain metastatic samples (G–H). The macrophage cap was associated with increased intracranial progression-free survival as determined by follow-up brain MRI (I) and extracranial progression-free survival as determined by follow-up PET-CT scans (J). The macrophage cap was also associated with increased response to systemic therapy, including chemotherapy and immunotherapy (K). Scale bars, 50 μm. *p* value <0.05 (∗), *p* value <0.01 (∗∗). The sample size, *n*, represents individual patient information from a minimum of three technical replicates per patient.

To further characterize the macrophage populations in the TME, we performed immunofluorescence staining across serial sections of brain metastatic specimens (high resolution in Figure 6, stitched whole slide images in Figure S7). Consistent with our transcriptomic data, we found enrichment of a population of CD163^+^, CD68^+^, MRC-negative macrophages in direct association with tumor cells at the leading edge of the brain metastases (Figures 6A–6C). These macrophages are the most common cell type in direct association with tumor cells, and the presence of this macrophage “cap” was associated with improved survival outcomes (Figures 6D–6F). We separately quantified macrophages within the tumor (not including the macrophage “cap”), and found, paradoxically, enrichment of macrophages associated with poor survival (<15 months, median 3.9 months, *n* = 10) compared to patients with good survival (>15 months, median 41 months, *n* = 9) (Figures 6G and 6H). In addition to worse overall survival, we found that the lack of a macrophage cap was associated with worse intracranial and extracranial progression-free survival of follow-up brain MRI and computed tomography scans with hazard ratios of 4.6 and 3.2, respectively (Figures 6I and 6J). These data suggest spatially distinct macrophage sub-types in brain metastases, which may have different impacts on tumor cell expansion and patient survival.

Finally, we explored whether the macrophage cap was associated with response to therapies. We examined intracranial disease as measured by the progression of disease on follow-up brain MRIs and found that brain metastatic specimens with macrophage caps were associated with improved intracranial progression-free survival in response to both radiation therapy (median survival 176 days without cap, median survival 1323 days with cap) and systemic therapies (median survival 192 days without cap, median survival 1272 days with cap) (Figures 6K and S8). Radiation therapy included a composite of patients receiving whole-brain radiation therapy and localized stereotactic radiosurgery. Systemic therapies included a composite of patients receiving chemotherapy, immunotherapy, and targeted therapy. Due to a lack of sample size in sub-groups, we were unable to assess responses specifically to HER2-targeted therapy (*n* = 6) or immunotherapy (*n* = 3). Notably, in the three patients who received immunotherapy, the one patient without a macrophage cap had evidence of disease progression within 1 month, while the two patients with a macrophage cap did not have any evidence of intracranial disease progression in the first year after resection (Table S1). Together, these data suggest that the recruitment of the identified macrophage population to the tumor-leading edge is associated with increased tumor responsiveness to therapeutic intervention, attenuated disease progression, and improved overall survival of patients harboring brain metastases.

### Reprogramming of intra-tumor macrophages is associated with poor survival outcomes in brain metastasis

**Figure 7:**
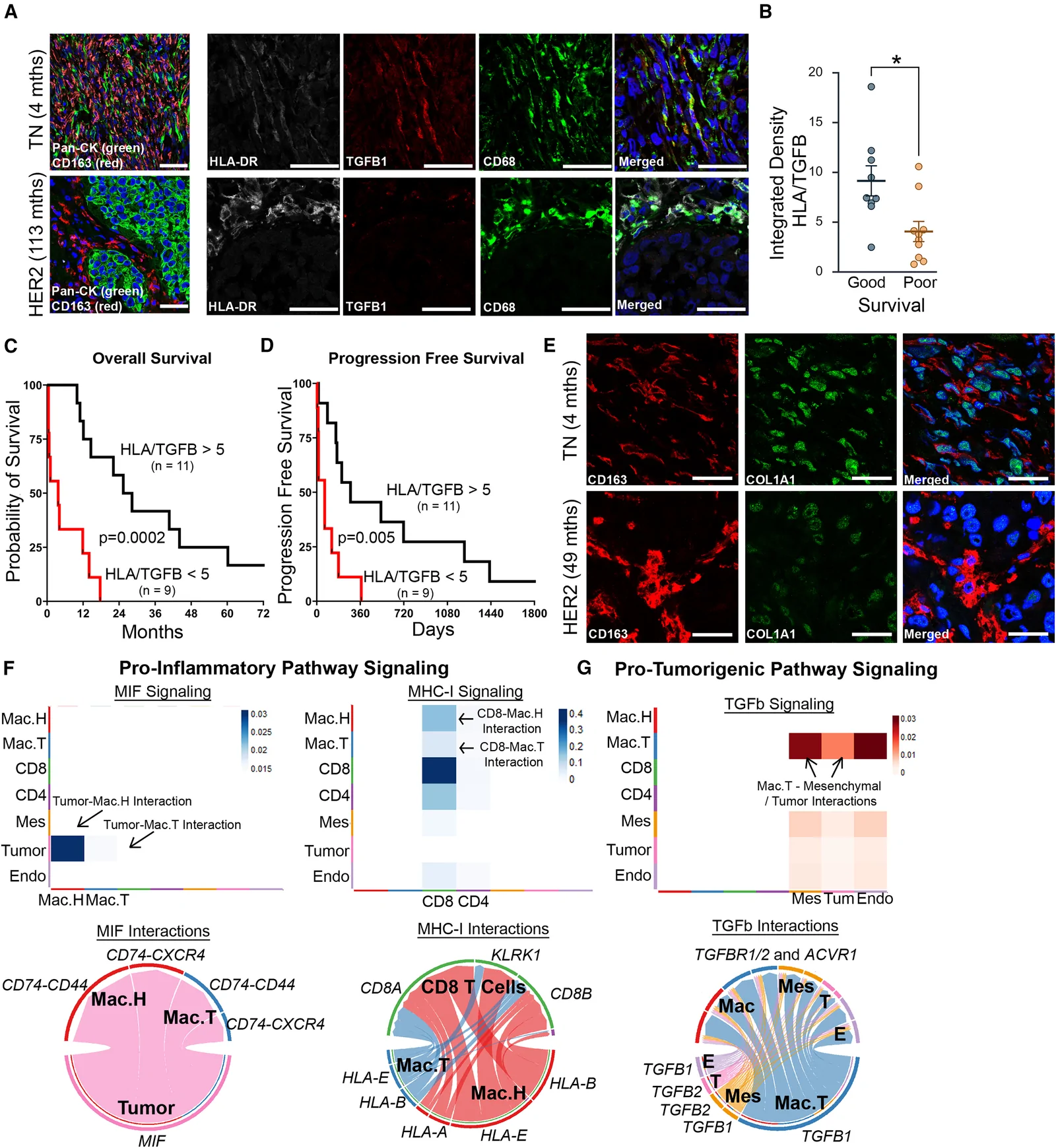
Inflammatory and pro-tumorigenic macrophage subtypes are associated with survival outcomes (A–D) Immunofluorescence staining of macrophages shows a change in macrophage morphology and function, specifically downregulation of inflammatory pathways (HLA-DR) and upregulation of pro-tumorigenic pathways (TGFB1), which is associated with poor survival outcomes. Log rank (Mantel-Cox) testing shows a statistically significant difference in overall and progression-free survival between tumors with macrophage caps and those without. Scale bars, 50 μm. *p* value <0.05 (∗). The sample size, *n*, represents individual patient information from a minimum of three technical replicates per patient. (E) Immunofluorescence staining shows abundant COL1A1 expression in macrophages in a TNBC specimen with poor survival compared to staining in macrophages in a HER2+ BC specimen with good survival. Scale bars, 25 μm. (F and G) CellChat predicts ligand receptor interactions between cell types, including CD8 and CD4 T cells, endothelial cells (Endo/E), tumor cells (Tum/T), mesenchymal cells (Mes), *HLA-DR*+ macrophages (Mac.H), and *TGFB1*+ macrophages (Mac.T), in the single nuclei dataset shows increased cell-cell communication probabilities via MIF and MHC-I pathway signaling between *HLA-DR*+ macrophages and tumor cells and cells in the tumor microenvironment. By contrast, there is increased cell-cell communication probability via TGFβ signaling between *TGFB1*+ macrophages and tumor cells and cells in the tumor microenvironment. Heatmaps are shown on the top, while chord diagrams with the specific ligand-receptor interaction driving communication probabilities are shown on the bottom.

To understand the potential mechanisms by which macrophages may promote or hinder tumor growth, we performed immunofluorescence staining for markers of macrophage function. Guided by the single nuclei pathway analysis results showing the upregulation of inflammatory pathways associated with improved survival and upregulation of pro-tumorigenic pathways such as TGFβ signaling in patients with poor survival (Figures 3F and 3G), we performed staining for MHC-II (HLA-DR) as a marker of inflammatory macrophage activation and TGFβ1 as a marker of a pro-tumorigenic macrophage phenotype (Figure 7A). In this regard, an immunosuppressive, *TGFB1*-high expressing macrophage population was recently identified in a spatial transcriptomic analysis in primary colorectal cancers.^19^ We found a significant increase in the HLA-DR/TGFβ1 ratio in patients with improved survival (>15 months, median 41 months, *n* = 9) compared to those with poor survival (<15 months, median 3.9 months, *n* = 10) (Figures 7B–7D and S8). In addition to the increased expression of TGFβ1, intra-tumor macrophages associated with poor survival also expressed high levels of the extracellular matrix protein, COL1A1, not seen in HLA-DR-high macrophages at the tumor boundary (Figure 7E).

### Changes in signaling pathways and cell-cell interactions in macrophage subtypes

To better define the distinct changes in signaling pathways and cell-cell interactions in HLA-DR-high and TGFβ1-high macrophage populations, we performed CellChat analysis on our single nuclei dataset. The CellChat v2 database includes receptors, ligands, and cofactors of over 2,000 heteromeric molecular complexes essential in known cell-cell interactions.^20^ The CellChat tool quantitatively infers and analyzes intercellular communication networks in single cell RNA-seq datasets and allows for predictions of signaling inputs and outputs for each sub-population of cells. CellChat analysis showed an increase in predicted macrophage migration inhibitory factor (MIF) signaling-mediated interactions between tumor cells and inflammatory HLA-DR-high macrophages, likely through tumor cell expression of *MIF* and macrophage expression of the cell surface receptor, *CD74,* and co-receptor, *CD44* (Figures 7F and 7G). MIF functions in a pro-inflammatory, chemokine-like mechanism for recruiting macrophages to sites of injury and infection.^21^ In addition, CellChat analysis also showed an increase in predicted HLA-DR-high macrophage to T cell interactions through MHC and complement signaling (Figures 7F and S9). MHC-mediated signaling represents a critical mechanism for the recruitment of T cells, leading to immune-mediated responses targeting cancer cells. Further support for this important macrophage-T cell interaction is provided by our single cell analysis of T cell populations showing that the upregulation of inflammatory response signaling is associated with good survival (Figure S3), and spatial transcriptomic data showing the enrichment of CD4^+^ T cells with macrophages at the tumor boundary (Figures 4, 5, and S5). These pathways represent putative mechanisms by which inflammatory, HLA-DR-high macrophages home to the tumor boundary and promote an immune response targeting brain metastases in patients with improved survival.

By contrast, CellChat analysis showed an increase in predicted *TGFB1*-high macrophage interactions with tumor and stromal cells, dependent on TGFβ and VEGF signaling (Figures 7D and S9). Increased macrophage expression of *VEGFA* has been previously associated with angiogenesis,^22^ and may be a potential mechanism by which TGFB1-high macrophages promote tumor growth and survival in patients with poor survival.

## Discussion

These studies identify spatially distinct tumor-macrophage interactions across breast cancer, lung cancer, and melanoma brain metastases. We identified distinct macrophage subsets, including inflammatory, HLA-DR-high macrophages at the tumor boundary and pro-tumorigenic, TGFβ1-high macrophages within the tumor. The distinct roles of these macrophages—whether promoting or inhibiting tumor growth and survival—appear to be context dependent, with changes in macrophage phenotype associated with survival outcomes.

Consistent with prior transcriptomic profiling of brain metastases,^12^^,^^13^^,^^14^^,^^16^ we identified pathways previously observed in brain metastases, including neural pathways, pathways promoting epithelial-mesenchymal transition, and immunomodulatory pathways. A major limitation of using single-cell transcriptomic analysis lies in sampling error when the spatial context is unavailable. This limitation is highlighted by our spatial transcriptomic data, where samples obtained from the leading edge of the tumor at the margins of resection (Figure 4) identified different macrophage populations compared to those present when sampling the tumor core (Figure S5). Even with advances in sequencing technology that allow for spatial resolution at the single cell level, the appropriate selection of tumor samples by considering histopathological and clinical characteristics remains critically important to identify distinct cell types that can elicit the differential regulation of metastatic tumors depending on spatial location. The use of Xenium and Visium spatial transcriptomic analyses allowed us to identify spatially distinct populations of macrophages, including a macrophage “cap” and intra-tumor macrophages, associated with different clinical outcomes. A recent publication on the Visium HD platform in the context of primary colorectal cancers observed macrophage enrichment at the primary tumor boundary and distinct pro-inflammatory and immunosuppressive (*TGFB1*+) macrophages.^19^ However, this study did not report whether changes in these macrophage populations were associated with clinical outcomes.

We characterized myeloid cell populations based on a combination of single-nuclei RNA profiling and cell-surface expression of canonical protein markers. Prior mouse studies have identified an important role for microglia in brain metastasis.^18^^,^^23^^,^^24^^,^^25^^,^^26^ However, although resident microglia and MDMs crossing the blood-brain barrier from circulation have many similarities in gene expression and function, we found that markers of microglia, including *P2RY12* and *SALL1*,^13^^,^^18^ were not expressed in most of the tumor-associated myeloid cells in our brain metastasis specimens. Our findings are consistent with prior studies in human brain metastasis^27^^,^^28^^,^^29^^,^^30^ which suggest that a majority of the myeloid cells in human brain metastases are derived from circulating MDMs, highlighting important differences between microenvironment cell types in mouse xenograft and primary human specimens. These findings raise the possibility that circulating monocytes may not only represent a biomarker for prognosis in patients with brain metastasis but also a target for systemic immunotherapies. Future studies are warranted to study circulating monocytes from blood samples of patients with brain metastases.

Several reports have identified distinct MDM populations in brain metastasis and sought to characterize MDM function using single cell RNA-seq analysis.^13^^,^^15^ However, the spatial distribution of the various macrophage populations and their association with patient survival were not interrogated in these studies. One study identifying two MDM populations in brain metastases: (1) *APOE*+ macrophages expressing markers of complement and MHC-II signaling and (2) *S100A8+* macrophages expressing *S100A* family genes, *CXCL8*, and low expression of MHC-II signaling.^15^ Others have reported that the enrichment of S100A8+/S100A9+ macrophages correlated with immunosuppression and decreased survival in patients with head and neck tumors.^31^ Interestingly, it was shown through the use of image mass cytometry of 46 brain metastases that macrophages expressing myeloperoxidase (*MPO*) are associated with long-term survival in patients with brain tumors.^32^ We found the enrichment of *APOE* but not *MPO* in the identified pro-inflammatory, HLA-DR+ macrophage in our dataset. However, given that *APOE* is also widely expressed at a low level throughout the brain parenchyma, we found MHC-II expression (encoded by the *HLA-DR* family of genes) to be a better marker for the pro-inflammatory macrophage population associated with improved survival. We also found the enrichment of *S100A*-gene family members in the intra-tumor, pro-tumorigenic, TGFβ-high macrophage population associated with poor survival. We denoted these macrophages as TGFβ-high macrophages based on the direct role of TGFβ signaling on their function.

Targeting tumor-associated macrophages in brain metastasis represents a promising target for immunotherapy strategies. The specific mechanisms leading to the reprogramming of these macrophages from inflammatory, HLA-DR+ macrophages to immunosuppressive macrophages remain unknown. Prior studies have associated changes in immunophenotyping with distinct genetic alterations in brain metastasis specimens.^33^ Pro-inflammatory macrophages have been previously identified in single-cell studies to be associated with improved patient outcomes in glioblastoma.^32^ Using CellChat analysis of the transcriptomic datasets generated from brain metastasis specimens, we identified MIF-CD74-mediated signaling as a putative mechanism by which MDMs home to the tumor boundary, and MHC-I and MHC-II mediated signaling by which inflammatory MDMs mediate the recruitment of immune cells, including T cells, to target brain metastases in patients with good survival. The MDM-T cell interaction has been previously noted in human brain metastasis.^12^ Consistent with these findings, our single-cell transcriptomic analysis showed the upregulation of inflammatory signaling in T cells in patients with good survival outcomes compared to poor survival outcomes, and with our spatial transcriptomic data showing the enrichment of CD4^+^ T cells with macrophages at the tumor boundary. Notably, in our dataset, T cells are greatly over-represented (76% of T cells) in the melanoma samples, particularly in patients with good responses to immunotherapies. Prior studies focused on T cell analysis, and subsets have implicated T cell immunophenotypes with survival outcomes.^34^

In contrast to pro-inflammatory signatures associated with good prognosis, we found specific signatures, including TGFβ1 signaling, associated with poor prognosis. TGFβ1 signaling in coordination with cytokines involved in type 3 inflammation has been previously shown to be important in the differentiation of monocytes into fibrogenic macrophages in the context of tissue repair and fibrosis.^35^ Brain metastatic tumors may usurp these mechanisms to evade the immune response,^36^ and upregulation of fibrogenic pathways in macrophages would lead to increased production of extracellular matrix proteins in brain metastases compared to primary tumors.^30^ In this regard, extracellular matrix-rich niches promote angiogenesis, proliferation, and immunosuppressive signaling in glioblastoma which was associated with increased phospho-AKT and phospho-ERK expression and enhanced signaling of those pathways at the matrix-stroma interface.^37^ Interestingly, multi-omics profiling of glioblastoma showed that therapy-induced glioblastoma regression induced by multiple therapies triggered a fibrotic response, and inhibition of treatment-induced fibrosis improved survival in pre-clinical murine models.^38^ Further studies are needed to evaluate the extent to which reprogramming of myeloid cells occurs in circulation prior to or following extravasation into the brain parenchyma.

The datasets we generated using multi-omic analyses of 23 brain metastasis specimens derived from patients with breast cancer, lung cancer, melanoma, and sarcoma brain metastases provide a rich resource for interrogating the expression of specific genes and transcriptional networks in distinct tumor types and associated TMEs in the brain parenchyma.

### Limitations of the study

Limitations of this study include the limited sample size, particularly spatial transcriptomic specimens (*n* = 12 Visium samples, *n* = 4 Xenium samples). Even with 30 total brain metastatic specimens, we were limited in sub-group analyses, such as the evaluation of outcomes with tumor sub-types as well as in evaluating specific responses to therapies, including chemotherapy, radiotherapy, targeted therapy, and immunotherapy, due to sample size. While our findings represent compelling associations between the spatially organized myeloid microenvironment and survival outcomes, further mechanistic studies using functional assays are needed to demonstrate definitive effects of macrophage phenotypes and macrophage-tumor interactions on tumor growth and response to therapies in the brain metastatic niche.

## Resource availability

### Lead contact

Requests for further information and resources should be directed to and will be fulfilled by the lead contact, Ann Marie Pendergast (ann.pendergast@duke.edu).

### Materials availability

This study did not generate new unique reagents.

### Data and code availability

All data associated with this study are present in the paper or the supplementary materials. The raw singl-cell RNA sequencing datasets are available at the NCBI Geo (GSE325196). Processed Seurat object files are available on Figshare (https://doi.org/10.6084/m9.figshare.30349819). This paper does not report original code. Any additional information required to re-analyze the data reported in this paper is available from the lead contact upon request.

## Acknowledgments

This work is the result of NIH funding, in whole or in part, and is subject to the NIH Public Access Policy. Through acceptance of this federal funding, the NIH has been given a right to make the work publicly available in PubMed Central. This work was supported by the 10.13039/100000005Department of Defense (W81XWH-22-1-0033 to A.P., W81XWH-22-1-0034 to C.K.A.), 10.13039/100000002National Institutes of Health (R01CA246133 to A.M.P., 1R38HL143612 to A.K., 5T32HL160494-03 to A.K., 1K38HL180998-01 to A.K., 1F31CA290692-01A1 to H.T., 5T32GM133352 to H.T., T32-CA009111 to A.T.), 10.13039/100007038Lung Cancer Research Foundation (A.M.P.), NCI Cancer Support Grant (P30-CA014236-47 to the Duke Cancer Institute supporting C.K.A. and A.M.P.), Translating Duke Health (C.K.A.), and NIH S10 grant (1S1-OD034340-01A1 for the Light Microscopy Core Facility). We thank the Duke University School of Medicine, Duke Cancer Institute, Translating Duke Health Initiative, and the Preston Robert Tisch Brain Tumor Center for the use of the Brain Tumor Center Biorepository (BTBR), which provided the Duke clinical specimens and pathology expertise. We would like to acknowledge the assistance of the Molecular Genomics Core at the Duke Molecular Physiology Institute, Duke University School of Medicine, for the generation of data for the manuscript (RRID:SCR_017860). We acknowledge the Duke Light Microscopy Core Facility for assistance with immunofluorescence experiments. The graphical abstract created in BioRender (https://BioRender.com/8njw7ki) is licensed under CC BY 4.0.

## Author contributions

Conceptualization and methodology: A.K., C.M.M., A.E.D.V., C.K.A., S.G.G., and A.M.P. data curation and formal analysis: A.K., C.M.M., A.E.D.V., V.J., H.T., A.T., J.J.G., C.K.A., S.G.G., and A.M.P. writing – original draft: A.K., C.M.M., and A.M.P. writing – review and editing: A.K., C.M.M., A.E.D.V., V.J., H.T., A.T., J.J.G., C.K.A., S.G.G., and A.M.P. funding acquisition: A.K., C.K.A., and A.M.P. supervision: S.G.G., C.K.A., and A.M.P.

## Declaration of interests

C.K.A. has consulted with Genentech, Eisai, IPSEN, Seattle Genetics, Astra Zeneca, Novartis, Immunomedics, Elucida, Athenex, and Roche; she has received research funding from PUMA, Lilly, Merck, Seattle Genetics, Nektar, Tesaro, G1-Therapeutics, ZION, Novartis, Pfizer, Astra Zeneca, Elucida, Caris, Incyclix, Beigene, and royalties from UptoDate.com and Jones and Bartlett. A.K., C.M.M., A.E.D.V., V.J., H.T., A.T., J.J.G., S.G.G., and A.M.P. have no conflicts to disclose.

## STAR★Methods

### Key resources table

**Table:** 

REAGENT or RESOURCE	SOURCE	IDENTIFIER
**Antibodies**		

Alpha Smooth Muscle Actin	BioLegend	Cat#904601; RRID: AB_2565041
CD163	Cell Signaling Technology	Cat#93498; RRID: AB_2800204
MRC1 (CD206)	BioLegend	Cat#321102; RRID: AB_571923
CD68	Abcam	Cat#ab955; RRID: AB_307338
COL1A1	Developmental Studies Hybridoma Bank (DSHB)	Cat#F1C3; RRID: AB_2827934
Cytokeratin (pan reactive)	BioLegend	Cat#682601; RRID: AB_439775
HLA-DR	Invitrogen	Cat#14-9956-82; RRID: AB_468639
TGF Beta 1	Proteintech	Cat#81746-2-RR; RRID: 3670503
Goat Anti-Mouse IgG2B	Jackson ImmunoResearch	Cat# 115-625-207
Goat Anti-Mouse IgG1	Jackson ImmunoResearch	Cat# 115-545-205
Goat Anti-Rabbit IgG	Jackson ImmunoResearch	Cat# 111-575-144

**Biological samples**		

Patient brain metastatic samples	Duke University School of Medicine BioRepository & Precision Pathology Center	https://brpc.duke.edu/

**Chemicals, peptides, and recombinant proteins**		

RNasin Plus Inhibitor	Promega	Cat#N2615
Optiprep Solution	Sigma	Cat#D1556
Normal Goat Serum	Jackson ImmunoResearch	Cat# 005-000-121
Paraformaldehyde	Sigma	Cat#441244
Triton X-100	Sigma	Cat#X100-5 ML
Fluoromount G with DAPI	ThermoFisher	Cat#00-4959-52

**Critical commercial assays**		

Chromium Single Cell 3′ Reagent Kits v2	10x Genomics	https://www.10xgenomics.com/support/universal-three-prime-gene-expression/documentation/steps/library-prep/assay-scheme-and-configuration-of-chromium-single-cell-3-v-2-libraries

**Deposited data**		

Single nuclei transcriptomic raw data	This paper	GSE325196
Visium spatial transcriptomic raw data	This paper	GSE325196
Xenium spatial transcriptomic raw data	This paper	GSE325196
Processed Seurat object files	This paper	https://doi.org/10.6084/m9.figshare.30349819

**Software and algorithms**		

BioRender	BioRender	https://www.biorender.com/
Cell Ranger (v5.0.1)	10x Genomics	https://www.10xgenomics.com/support/software/cell-ranger/latest
Fast Gene Set Enrichment Analysis (FGSEA)	Bioconductor	https://bioconductor.org/packages/release/bioc/html/fgsea.html
Fiji	NIH	https://imagej.net/software/fiji/downloads
Prism 10 (v9.4.0)	GraphPad	https://www.graphpad.com/
Loupe Browser (v5.0.1)	10x Genomics	https://www.10xgenomics.com/support/software/loupe-browser/latest
R Statistical Software (v4.4.0)	The R Project	https://www.r-project.org/
Seurat (v5.1.0)	Satija Lab^39^	https://satijalab.org/seurat/articles/install_v5.html
SeuratExtend	Hua et al.^40^	https://github.com/huayc09/SeuratExtend
SpatialeXpressionR (spacexr)	Bioconductor	https://www.bioconductor.org/packages/release/bioc/html/spacexr.html
Space Ranger	10x Genomics	https://www.10xgenomics.com/support/software/space-ranger/latest
CellChat	Jin et al.^20^	https://github.com/sqjin/CellChat

### Experimental model and study participant details

#### Study design and subjects

The main objective of the study was to compare the tumor and microenvironment cell populations across human lung, breast, melanoma, and sarcoma brain metastases. We prospectively collected and analyzed 30 samples from patients with brain metastases of which 23 samples passed quality control for single nuclei sequencing analysis (*n* = 11 lung cancer, *n* = 8 breast cancer, *n* = 3 melanoma, *n* = 1 sarcoma). Ten samples were submitted for Visium spatial transcriptomic analysis. Four samples were submitted for Xenium spatial transcriptomic analysis. Protein validation with immunofluorescence staining was completed on 22 samples. Details on patient clinical characteristics, including sex, age, ethnicity, race, tumor site, and histopathologic diagnosis are provided in Table S1.

#### Study approval

The human biological samples and associated clinical data in this study were collected and consented at Duke University Medical Center through an approved biobanking protocol (Pro00007434) and analyzed under exempt protocols (Pro00109526, Pro00103267, and Pro00104321) after review and approval/exemption by the Duke University Health System Institutional Review Board. Patients consented to donating brain metastasis specimens to the biobanking protocol for research use.

### Method details

#### Human brain metastases preparation for single nuclei sequencing

Frozen tissue samples were obtained from brain resections at Duke University Medical Center from 1998 to 2022. The tissue was thawed and minced on ice prior to transfer to 1 mL of lysis buffer (10 μL 1M Tris-HCl, pH 7.5, 1 μL 5M NaCl, 3 μL 1M MgCl_2_, 10 μL 10% NP-40, 1 μL RNasin Plus (Promega N2615) in sterile water). The sample was incubated on ice for 5 min and then triturated 10–15 times every 30 s for 10 min. The sample was strained through a 70 μm filter and centrifuged at 300*g* for 5 min at 4°C. The cell pellet was resuspended into 1.5 mL Optiprep solution (Sigma D1556) and centrifuged at 10,000*g* for 10 min at 4°C. The cell pellet was resuspended in 1 mL resuspension buffer (1X PBS, 1% BSA, RNase Plus inhibitor (Promega N2615)) and filtered using a 40 μm filter. Finally, the suspension was centrifuged again at 10,000*g* for 10 min at 4°C and resuspended in resuspension buffer for submission to the Duke Molecular Genomics Core for single nuclei sequencing. Single nuclei were prepared for cDNA amplification and library preparation using Single Cell 3′ Reagent Kits v2 (10x Genomics) at the Molecular Genomics Core facility at the Duke Molecular and Physiology Institute (DMPI). The cDNA libraries were sequenced on the NovaSeq 6000 SP and S1 platforms by the Duke Center for Genomic and Computational Biology core facility.

#### Human brain metastases single nuclei sequencing data processing

The primary analytical pipeline for single nuclei sequencing analysis followed the recommended protocols from 10X Genomics. Briefly, Cell Ranger v5.0.1 was used to demultiplex raw FASTQ files, align the reads to the 10x GRCh38 reference transcriptome, and generate gene expression matrices for all single cells in each sample. Further downstream analysis was performed in R v4.4.0. The R package, Seurat v5.1.0,^41^ was used for quality control and further downstream analysis on the feature-barcode matrices produced by Cell Ranger. The top 3,000 genes were selected based on variance using the Seurat implementation FindVariableFeatures. These genes were then centered and scaled using Seurat’s implementation, sctransform v2, and percent mitochondrial genes as a variable to regress out (percent_mt = 0.10). Normalized and scaled data were subsequently used for principal components analysis (npcs = 100), and cell cluster analysis was performed using Seurat’s FindNeighbors and FindClusters functions. Visualization was performed using uniform manifold approximation and projection. Pathway analysis and gene set enrichment analysis was performed using the R packages, SeuratExtend^40^ and fgsea, which use Wilcoxon rank-sum test and Bonferroni correction to generate adjusted *p* values for each gene.

#### Visium spatial transcriptomic analysis

H&E staining of OCT and formalin-fixed and paraffin embedded (FFPE) brain metastases resections was performed, and samples underwent formal histopathologic review for tumor grade, percent necrosis, and percent glial cells. 6 frozen samples and 6 FFPE samples were chosen to proceed to Visium transcriptomic analysis (Table S1) using sample processing and data analysis pipelines that are previously described.^42^ Briefly, 10 μm sections were cut from frozen OCT blocks or FFPE blocks and mounted onto Visium spatial gene expression slides and processed according to the manufacturer’s protocol (10x Genomics). Tissue sections were permeabilized immediately to capture RNA molecules onto barcoded spots on the slide. Libraries were then prepared and sequenced on the NovaSeq 6000 SP platform by the Duke Center for Genomic and Computational Biology core facility.

Fiducial 50 μm spots on the H&E image were manually aligned using Loupe browser v5.0.1 (10x Genomics). Space Ranger software (10x Genomics) was used to process raw FASTQ files, align the reads to the 10x GRCh38 reference transcriptome, and generate gene expression matrices for all single cells in each sample. Further downstream analysis of the spot UMI table and reference H&E section was performed in R using Seurat v4.1.0. Data was normalized using sctransform, and spatial gene expression was visualized using the Seurat’s SpatialFeaturePlot function.

#### Xenium spatial transcriptomics analysis

Xenium spatial transcriptomic analysis was conducted using the Xenium Analyzer at the Duke Molecular Physiology Institute as previously described.^43^ Briefly, four frozen brain metastasis specimens chosen to proceed to Xenium transcriptomic analysis were processed using the Xenium Analyzer, which performs automated image preprocessing, transcript decoding, and cell segmentation based on DAPI-stained nuclei. The resulting outputs, including the feature-cell matrix, transcript coordinates, and cell boundary files, were imported into R (v4.3.1) using the LoadXenium function from Seurat v5.^39^ Data were loaded and filtered using an nFeature_Xenium>5 and nCount_Xenium>10. Spatial deconvolution was performed using spacexr (v2.2.1) with a custom single-cell reference. Differential gene expression was assessed using the Wilcoxon test on SCTransform-normalized data via Seurat’s FindMarkers function.

#### Immunofluorescence

5 μm frozen OCT embedded tissue sections were thawed and fixed with 4% PFA. Sections were permeabilized in 0.1% Triton X- in 3% goat serum in 1x TBS for 15 min and blocked in 3% goat serum in TBS with 0.05% Tween 20 for 30 min. Sections were incubated with primary antibodies to CD163 (1:250 Cell Signaling 93498), HLA-DR (1:100 Invitrogen 14-9956-82), pan-CK (1:250 Biolegend 682602), smooth muscle actin (1:250 Biolegend 904601), TGFβ1 (1:100 Proteintech 81746-2-RR), CD68 (1:100 Abcam AB955), and COL1A1 (1:30 DHSB F1C3) overnight at 4°C in a humidified chamber. Sections were then washed with 1x TBS-T followed by incubation with secondary antibody at 1:250 dilution in blocking solution for 1 h at room temperature. Sections were then washed with 1x TBS-T and mounted using aqueous mounting media containing DAPI counter-stain.

Cell imaging was performed on the Leica SP8 confocal microscope at the Duke Light Microscope Core Facility. Quantification of macrophage counts was conducted in a strictly ‘blinded’ manner where none of the personnel were provided patient information or clinical outcome data (all samples were de-identified for analysis) until after all the quantification was completed. Quantification was performed across all cells in each section using particle analysis function in Fiji software. A subset (>3 distinct views) of high-resolution images was used for quantification of the intra-tumor macrophages. Replicate images from each sample were aggregated with the mean used to define patient-level macrophage phenotype for overall survival and progression-free survival analyses. For representative high-resolution images, linear scale adjustments were applied. Representative stitched confocal images of entire tumor section are provided (Figure S7).

### Quantification and statistical analysis

The number of patient samples for each experiment is indicated in the figure legends. Data aggregation and statistical analysis for immunofluorescence studies was performed using GraphPad Prism (v9.4.0) while graphs were generated using BioRender. All graphs are represented as means with standard error of measurement, and a two-tailed *p*-value of less than 0.05 was considered significant. Power calculations were used to determine the necessary sample size for all immunofluorescence experiments based on effect size and results observed in the single nuclei RNA sequencing data. Differences in continuous variables between two groups were assessed using the unpaired parametric *t* test or Wilcoxon rank test. Comparisons between more than two groups were assessed using analysis of variance (ANOVA). Post-hoc Tukey testing for multiple comparisons tests was only performed when statistical significance (*p* < 0.05) was identified from the ANOVA. Survival analysis was completed using Log rank (Mantel-Cox) testing. For all statistical analyses, *n* represents individual patient specimen. ∗, ∗∗, and ∗∗∗ denote *p*-values of <0.05, <0.01, and <0.001, respectively.

## Supplemental information

Document S1. Figures S1–S9, Tables S2 and S3

Table S1. Human brain metastasis patient characteristics, related to Figure 1
