# Supplementary material for: Spatially resolved transcriptomics in human brain metastases identifies macrophage-tumor interactions associated with survival
Source: iScience. 2026 Aug 12;29(8):116517. doi: 10.1016/j.isci.2026.116517 (PMC13523839; doi:10.1016/j.isci.2026.116517)
Supplement: Supplementary file 1 [file mmc1.pdf]

iScience, Volume *29*

Supplemental information

Spatially resolved transcriptomics in human brain metastases identifies macrophage-tumor interactions associated with survival

Aaditya Khatri, Courtney M. McKernan, Amanda E.D. Van Swearingen, Vaibhav Jain, Hannah L. Thrash, Arianna Towne, Jing Jin Gu, Simon G. Gregory, Carey K. Anders, and Ann Marie Pendergast

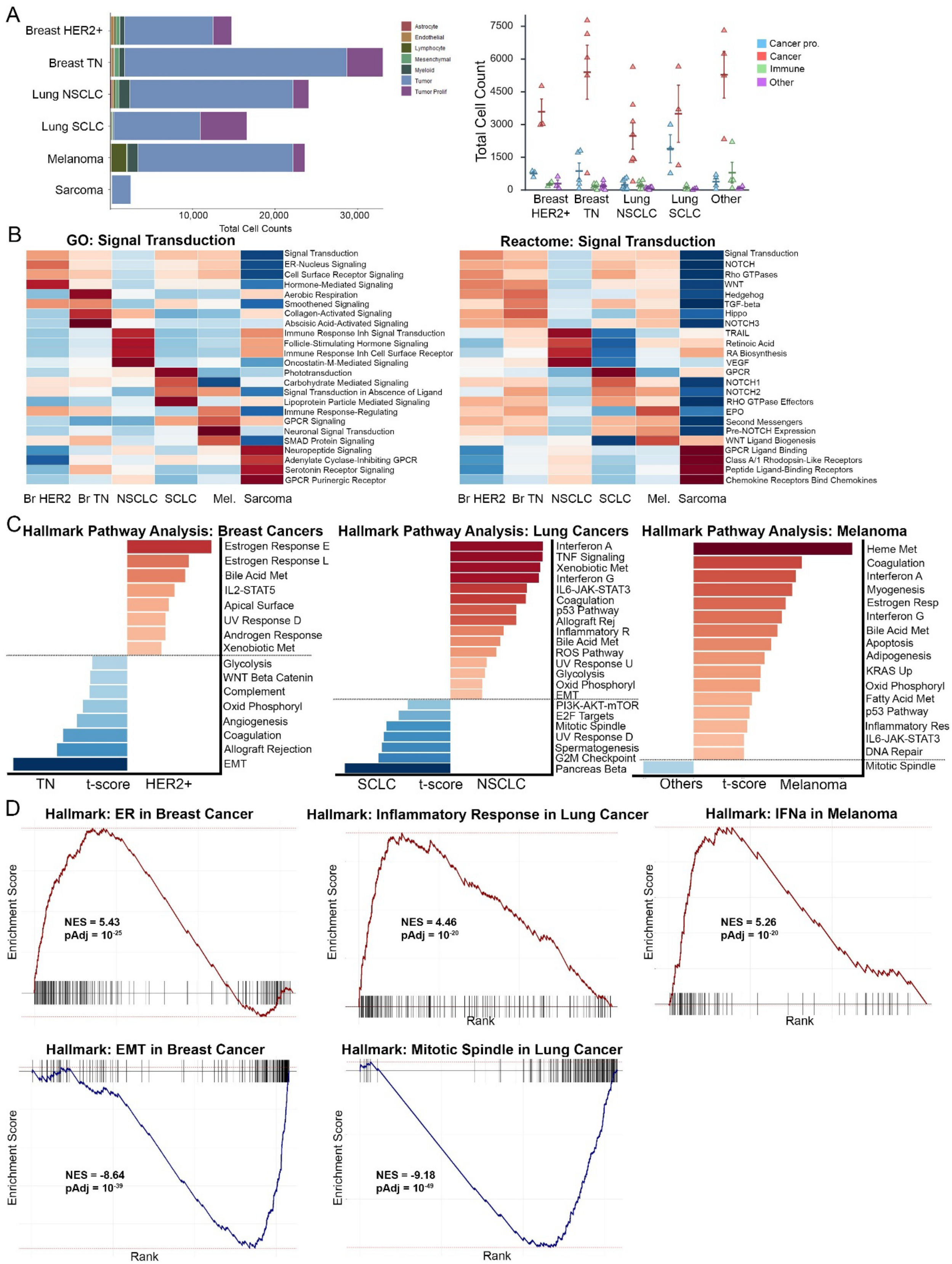
A
Breast HER2+
Breast TN
Lung NSCLC
Lung SCLC
Melanoma
Sarcoma
10,000
20,000
30,000
Total Cell Counts
Astrocyte
Endothelial
Lymphocyte
Mesenchymal
Myeloid
Tumor
Tumor Prolif
Total Cell Count
7500
6000
4500
3000
1500
0
Breast HER2+
Breast TN
Lung NSCLC
Lung SCLC
Other
Cancer pro.
Cancer
Immune
Other
B
GO: Signal Transduction
Signal Transduction
ER-Nucleus Signaling
Cell Surface Receptor Signaling
Hormone-Mediated Signaling
Aerobic Respiration
Smoothened Signaling
Collagen-Activated Signaling
Abscisic Acid-Activated Signaling
Immune Response Inh Signal Transduction
Follicle-Stimulating Hormone Signaling
Immune Response Inh Cell Surface Receptor
Oncostatin-M-Mediated Signaling
Phototransduction
Carbohydrate Mediated Signaling
Signal Transduction in Absence of Ligand
Lipoprotein Particle Mediated Signaling
Immune Response-Regulating
GPCR Signaling
Neuronal Signal Transduction
SMAD Protein Signaling
Neuropeptide Signaling
Adenylate Cyclase-Inhibiting GPCR
Serotonin Receptor Signaling
GPCR Purinergic Receptor
Br HER2 Br TN NSCLC SCLC Mel. Sarcoma
Reactome: Signal Transduction
Signal Transduction
NOTCH
Rho GTPases
WNT
Hedgehog
TGF-beta
Hippo
NOTCH3
TRAIL
Retinoic Acid
RA Biosynthesis
VEGF
GPCR
NOTCH1
NOTCH2
RHO GTPase Effectors
EPO
Second Messengers
Pre-NOTCH Expression
WNT Ligand Biogenesis
GPCR Ligand Binding
Class A/1 Rhodopsin-Like Receptors
Peptide Ligand-Binding Receptors
Chemokine Receptors Bind Chemokines
Br HER2 Br TN NSCLC SCLC Mel. Sarcoma
C
Hallmark Pathway Analysis: Breast Cancers
Estrogen Response E
Estrogen Response L
Bile Acid Met
IL2-STAT5
Apical Surface
UV Response D
Androgen Response
Xenobiotic Met
Glycolysis
WNT Beta Catenin
Complement
Oxid Phosphoryl
Angiogenesis
Coagulation
Allograft Rejection
EMT
TN t-score HER2+
Hallmark Pathway Analysis: Lung Cancers
Interferon A
TNF Signaling
Xenobiotic Met
Interferon G
IL6-JAK-STAT3
Coagulation
p53 Pathway
Allograft Rej
Inflammatory R
Bile Acid Met
ROS Pathway
UV Response U
Glycolysis
Oxid Phosphoryl
EMT
PI3K-AKT-mTOR
E2F Targets
Mitotic Spindle
UV Response D
Spermatogenesis
G2M Checkpoint
Pancreas Beta
SCLC t-score NSCLC
Hallmark Pathway Analysis: Melanoma
Heme Met
Coagulation
Interferon A
Myogenesis
Estrogen Resp
Interferon G
Bile Acid Met
Apoptosis
Adipogenesis
KRAS Up
Oxid Phosphoryl
Fatty Acid Met
p53 Pathway
Inflammatory Res
IL6-JAK-STAT3
DNA Repair
Mitotic Spindle
Others t-score Melanoma
D
Hallmark: ER in Breast Cancer
Enrichment Score
NES = 5.43
pAdj = 10^-25
Hallmark: Inflammatory Response in Lung Cancer
Enrichment Score
NES = 4.46
pAdj = 10^-20
Rank
Hallmark: IFNa in Melanoma
Enrichment Score
NES = 5.26
pAdj = 10^-20
Rank
Hallmark: EMT in Breast Cancer
Enrichment Score
NES = -8.64
pAdj = 10^-39
Rank
Hallmark: Mitotic Spindle in Lung Cancer
Enrichment Score
NES = -9.18
pAdj = 10^-49
Rank

Figure S1. Cell Count and Pathway Analysis in Cancer Sub-Types. (A) Distribution of cell counts by cell type across cancer type. (B) GO and Reactome pathway analysis showing pathways enriched in each tumor cell type. (C) Hallmark pathway analysis showing upregulated pathways within breast (HER2 vs triple negative), lung (NSCLC vs SCLC) subtypes as well as in melanoma compared to other cancers. (D) GSEA showing upregulation or downregulation of selected pathways from (C) in breast cancer, lung cancer, and melanoma.

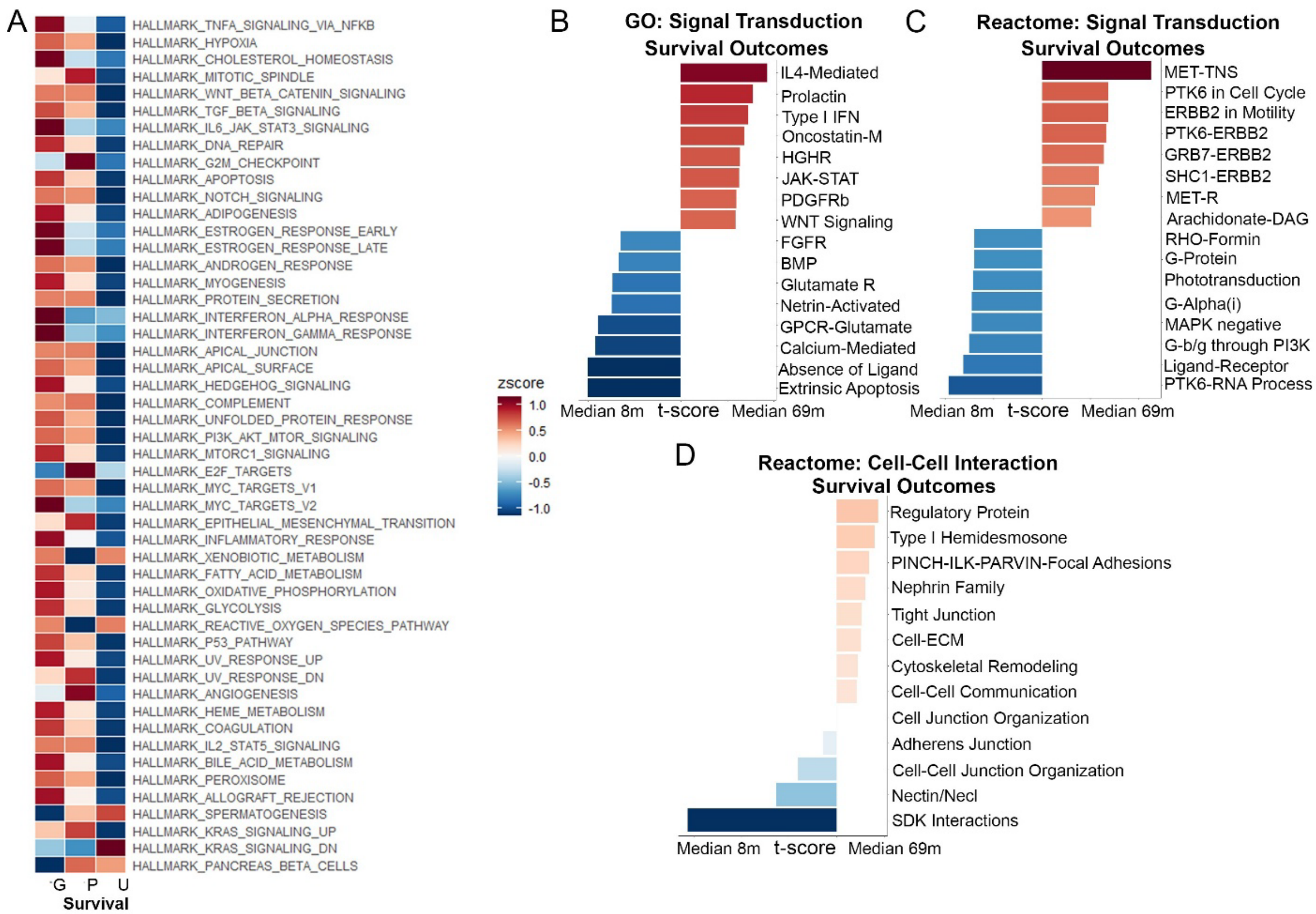

Figure S2. Pathway analysis based on patient survival outcomes. (A) Hallmark pathway analysis showing all 40 pathways in good (G), poor (P), and unknown (U) survival groups. (B-D) GO, Reactome Signal Transduction, and Reactome Cell-Cell Interaction pathway analyses identified enrichment of inflammatory pathways in patients with good survival (>24 months (n=9)) compared to poor survival (<24 months (n = 10)).

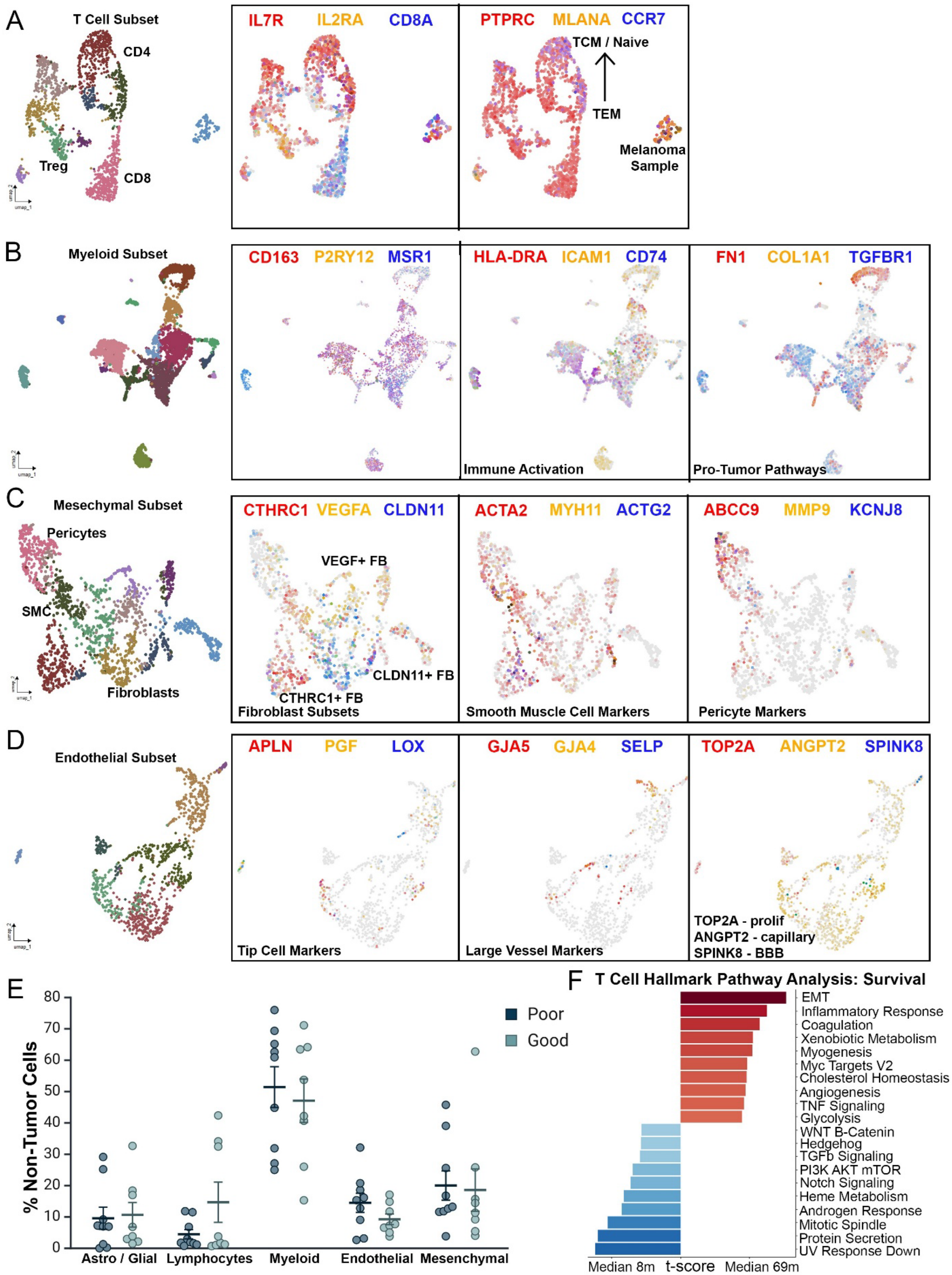

A
T Cell Subset
CD4
Treg
CD8
IL7R IL2RA CD8A
PTPRC MLANA CCR7
TCM / Naive
TEM
Melanoma Sample
B
Myeloid Subset
CD163 P2RY12 MSR1
HLA-DRA ICAM1 CD74
FN1 COL1A1 TGFBR1
Immune Activation
Pro-Tumor Pathways
C
Mesechymal Subset
Pericytes
SMC
Fibroblasts
CTHRC1 VEGFA CLDN11
VEGF+ FB
CLDN11+ FB
CTHRC1+ FB
Fibroblast Subsets
ACTA2 MYH11 ACTG2
Smooth Muscle Cell Markers
ABCC9 MMP9 KCNJ8
Pericyte Markers
D
Endothelial Subset
APLN PGF LOX
Tip Cell Markers
GJA5 GJA4 SELP
Large Vessel Markers
TOP2A ANGPT2 SPINK8
TOP2A - prolif
ANGPT2 - capillary
SPINK8 - BBB
E
% Non-Tumor Cells
0 10 20 30 40 50 60 70 80
Astro / Glial
Lymphocytes
Myeloid
Endothelial
Mesenchymal
Poor
Good
F
T Cell Hallmark Pathway Analysis: Survival
EMT
Inflammatory Response
Coagulation
Xenobiotic Metabolism
Myogenesis
Myc Targets V2
Cholesterol Homeostasis
Angiogenesis
TNF Signaling
Glycolysis
WNT B-Catenin
Hedgehog
TGFb Signaling
PI3K AKT mTOR
Notch Signaling
Heme Metabolism
Androgen Response
Mitotic Spindle
Protein Secretion
UV Response Down
Median 8m
t-score
Median 69m

Figure S3. Sub-cluster analysis and quantification of microenvironment cell types in 23 brain metastatic samples. (A-D) UMAP projection and gene expression of markers used to characterize T cell (A), myeloid (B), mesenchymal (C), and endothelial (D) cell sub-types. (E). Two-way ANOVA followed by post-hoc Tukey testing showed no significant difference in microenvironment cell proportions when comparing patients with good survival (>20 months, median 69 months) vs poor survival (<20 months, median 8 months) outcomes. (F) Hallmark pathway analysis within T cells showing the most up- and down-regulated pathways in good and poor survival groups.

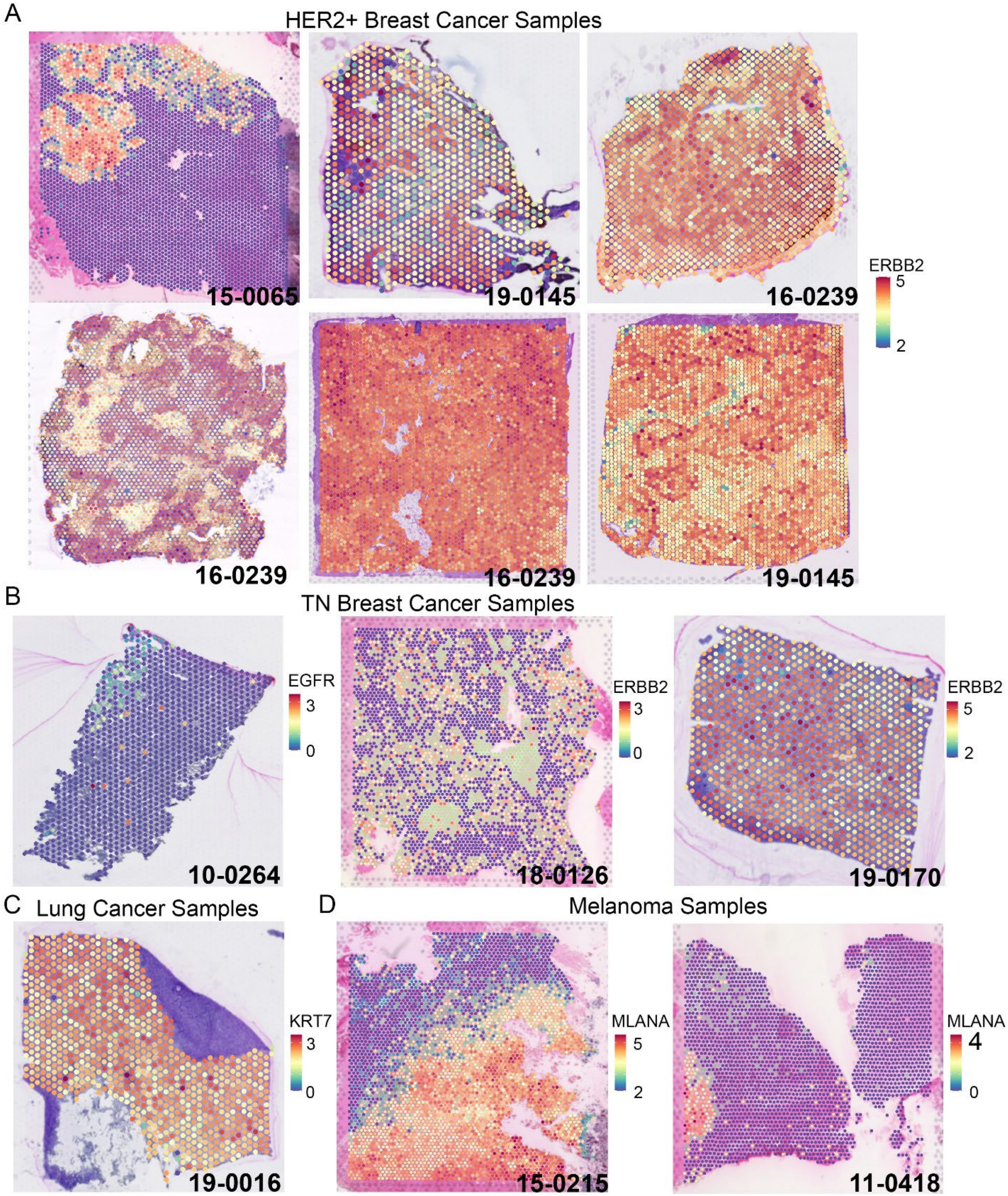

Figure S4. Visium spatial transcriptomic analysis in brain metastatic samples. (A-D) Feature plot showing distribution of cancer cells (*ERBB2, EGFR, KRT7,* or *MLANA*) in all 10 Visium spatial transcriptomic samples. Note, 3 out of the 12 images shown are from samples that were sequenced in replicates from the same patient (16-0239, 19-0145).

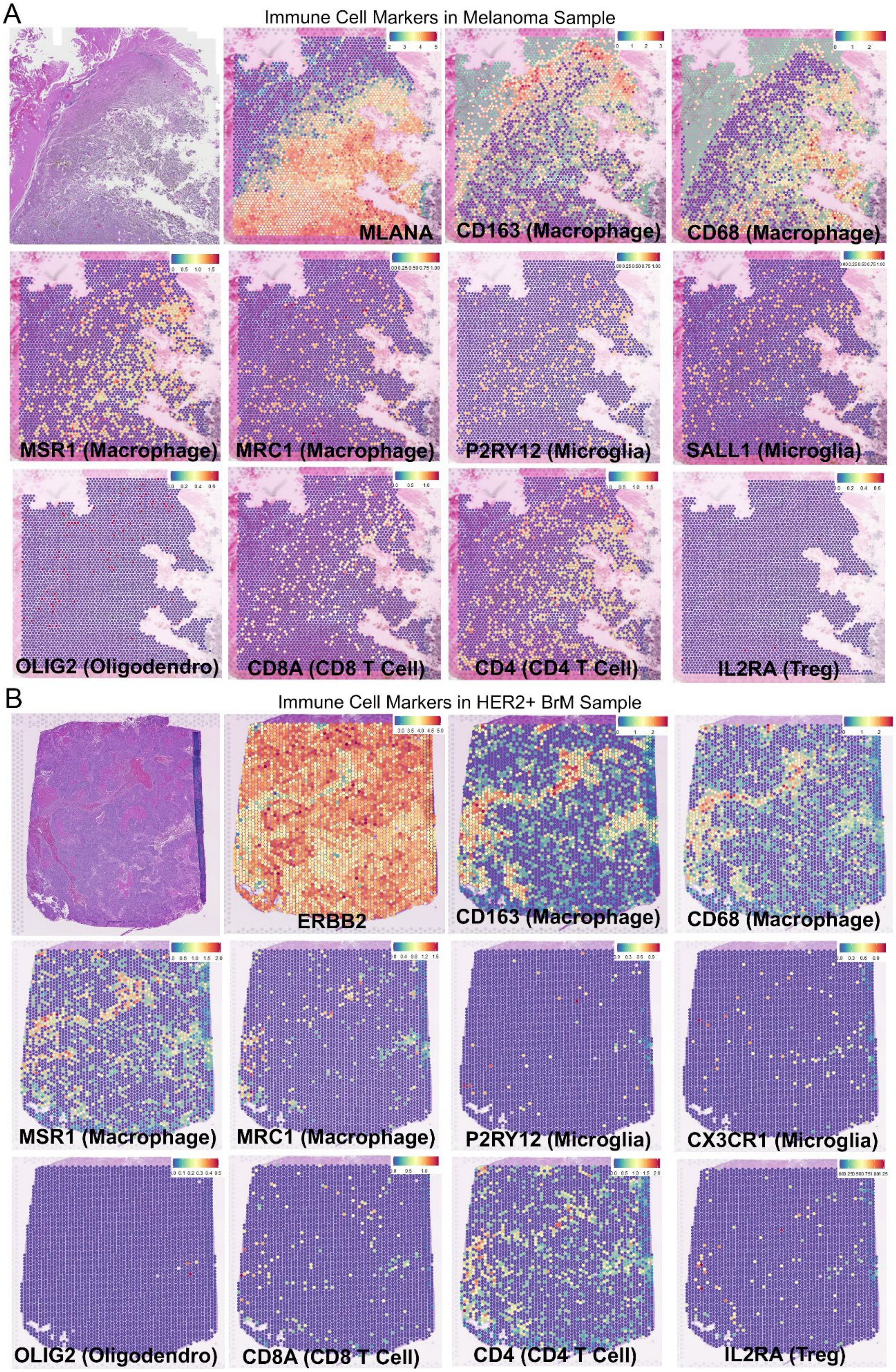

A
Immune Cell Markers in Melanoma Sample
MLANA
CD163 (Macrophage)
CD68 (Macrophage)
MSR1 (Macrophage)
MRC1 (Macrophage)
P2RY12 (Microglia)
SALL1 (Microglia)
OLIG2 (Oligodendro)
CD8A (CD8 T Cell)
CD4 (CD4 T Cell)
IL2RA (Treg)
B
Immune Cell Markers in HER2+ BrM Sample
ERBB2
CD163 (Macrophage)
CD68 (Macrophage)
MSR1 (Macrophage)
MRC1 (Macrophage)
P2RY12 (Microglia)
CX3CR1 (Microglia)
OLIG2 (Oligodendro)
CD8A (CD8 T Cell)
CD4 (CD4 T Cell)
IL2RA (Treg)

Figure S5. Visium spatial transcriptomic analysis identifies enrichment of macrophages at the leading edge of the tumor. (A-B) Feature plot showing enrichment of a macrophage subset as defined by expression of *CD163* and *CD68* compared to lymphocytes (*CD8A*, *CD4, IL2RA*), oligodendrocytes (*OLIG2*), and microglia (*P2RY12, SALL1, CX3CR1*) at the leading edge of the tumor in the melanoma (A) and HER2+ breast cancer (B) brain metastatic Visium spatial transcriptomic samples.

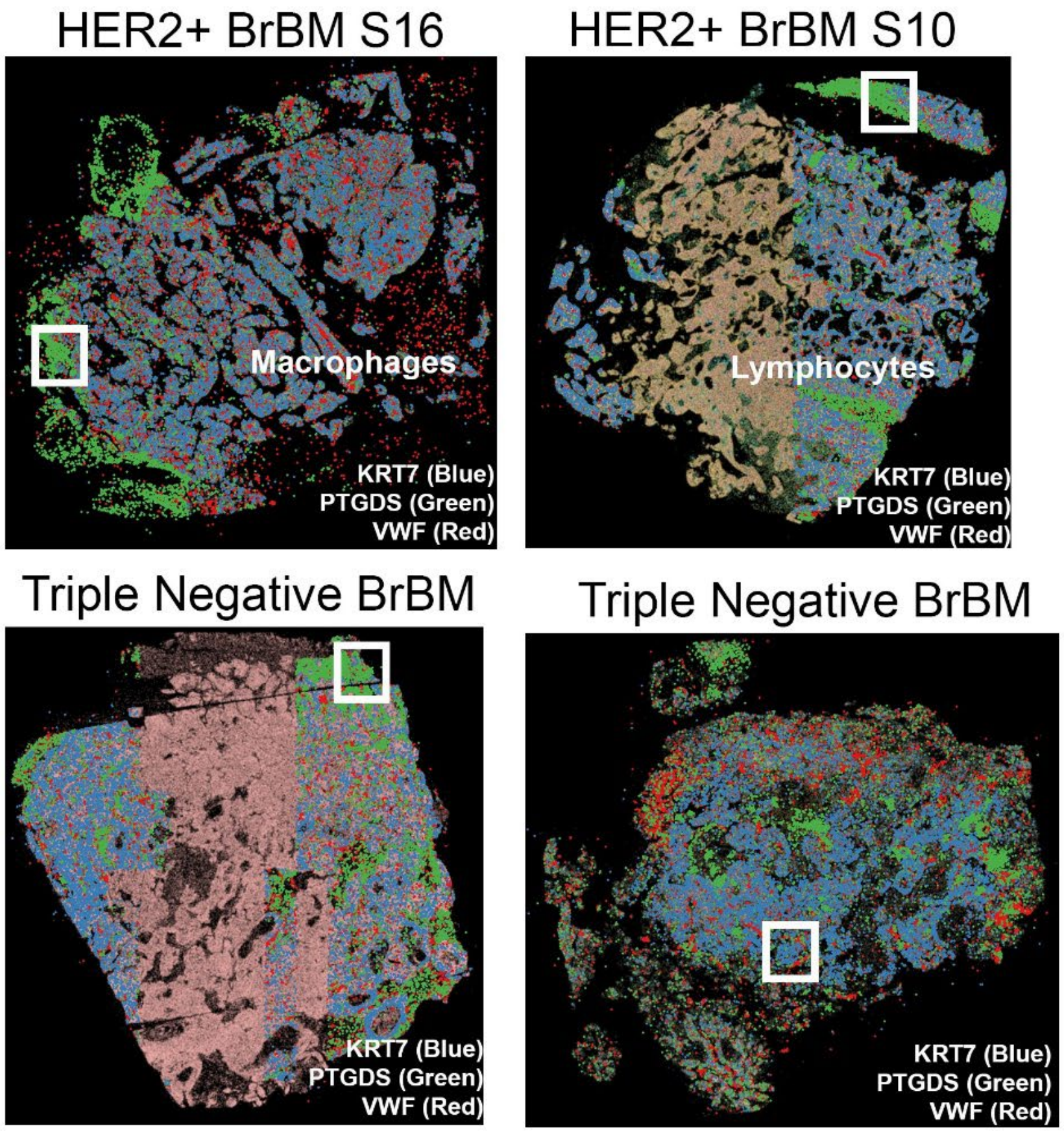

Figure S6. Xenium spatial transcriptomic analysis of four brain metastatic breast cancer samples. Distribution of tumor cells (*KRT7*, blue), brain parenchyma (*PTGDS*, green), and endothelial cells (*VWF*, red) across all four samples submitted for Xenium spatial transcriptomic analysis. The white boxes denote the regions shown in main figure 5A.

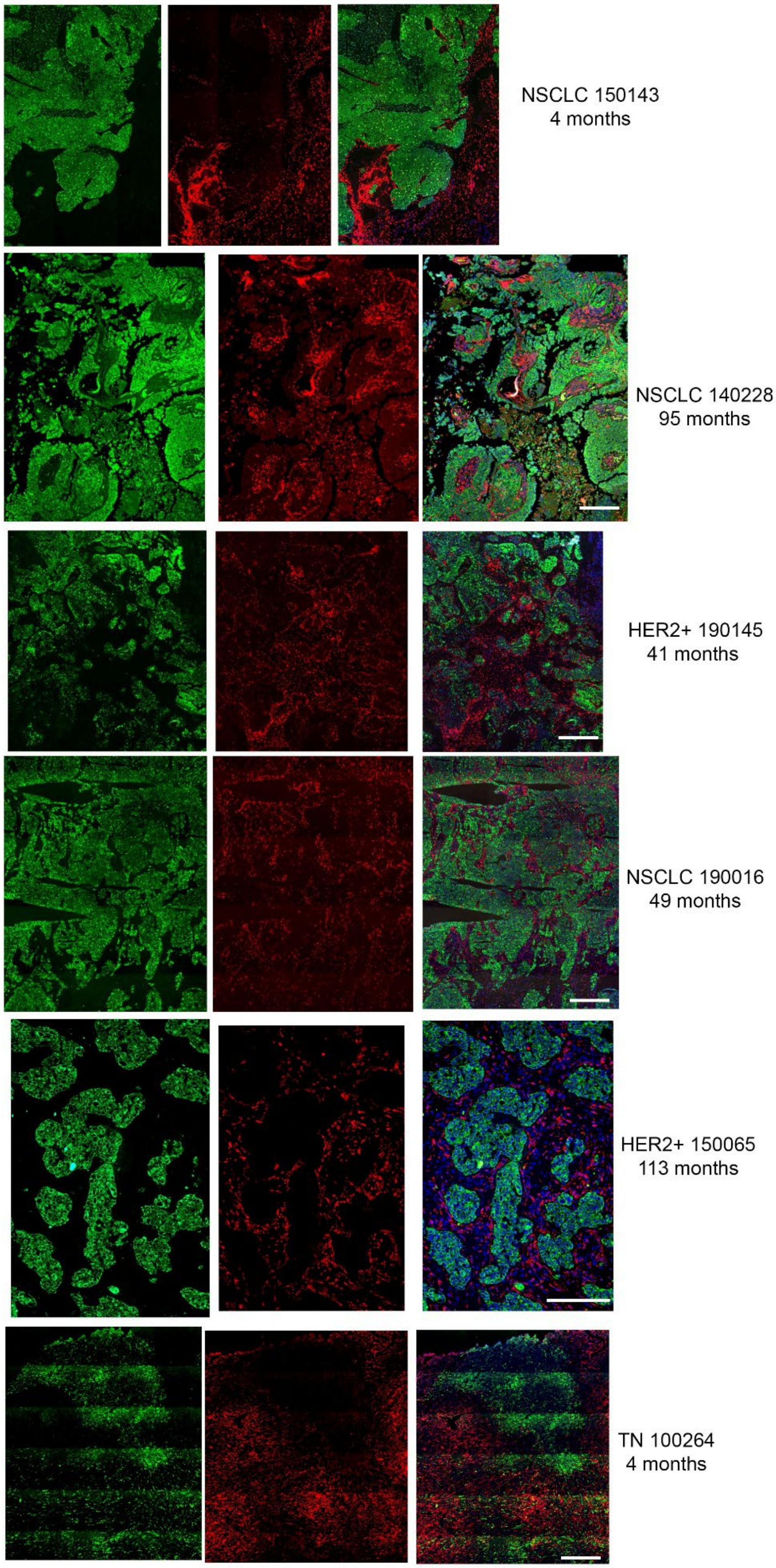
NSCLC 150143
4 months
NSCLC 140228
95 months
HER2+ 190145
41 months
NSCLC 190016
49 months
HER2+ 150065
113 months
TN 100264
4 months

Figure S7. Whole-section stitched immunofluorescence staining of tumor cells and macrophages. Whole slide stitched images from which high-resolution images were generated in Figure 6. Pan CK (tumor) is shown in green. CD163 (macrophages) is shown in red. Scale bar = 500μm.

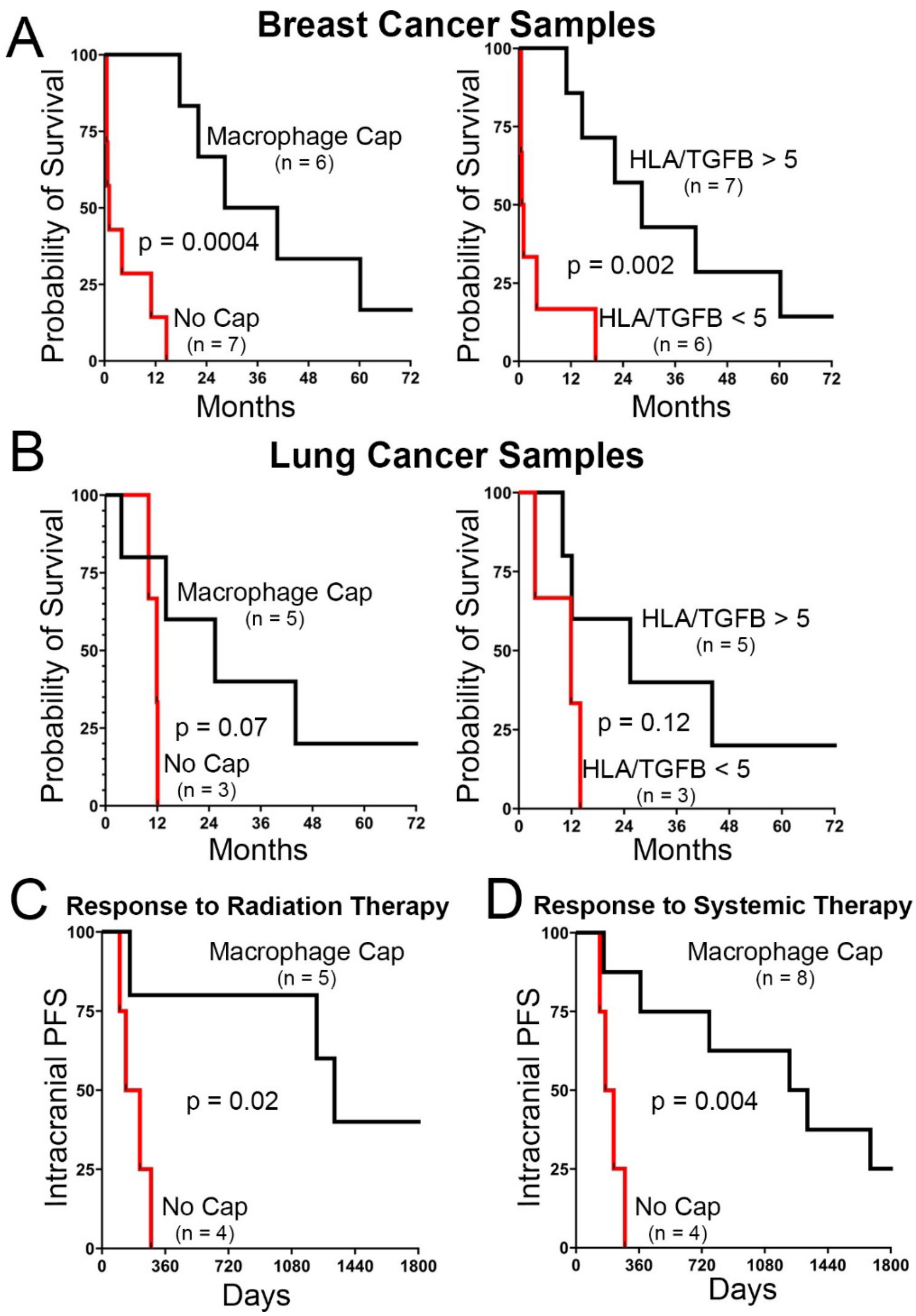

Figure S8. Sub-group analysis of clinical outcomes in breast and lung cancer. (A) Enrichment of macrophages at the tumor leading edge – a “macrophage cap” – and pro-inflammatory macrophage signaling as measured by HLA/TGFB ratio are associated with a statistically significant improvement in survival across breast cancer brain metastatic samples. (B) Overall survival analysis in lung cancer patients. (C-D) Intracranial response to radiation (C) or systemic therapy (D) as determined by progression of disease on follow up brain MRI scans in patients with breast and lung cancers. Brain metastatic samples with macrophage caps were associated with improved intracranial disease progression free survival in response to both radiation and systemic therapies. Overall survival analysis was conducted with log-rank (Mantel-Cox) testing.

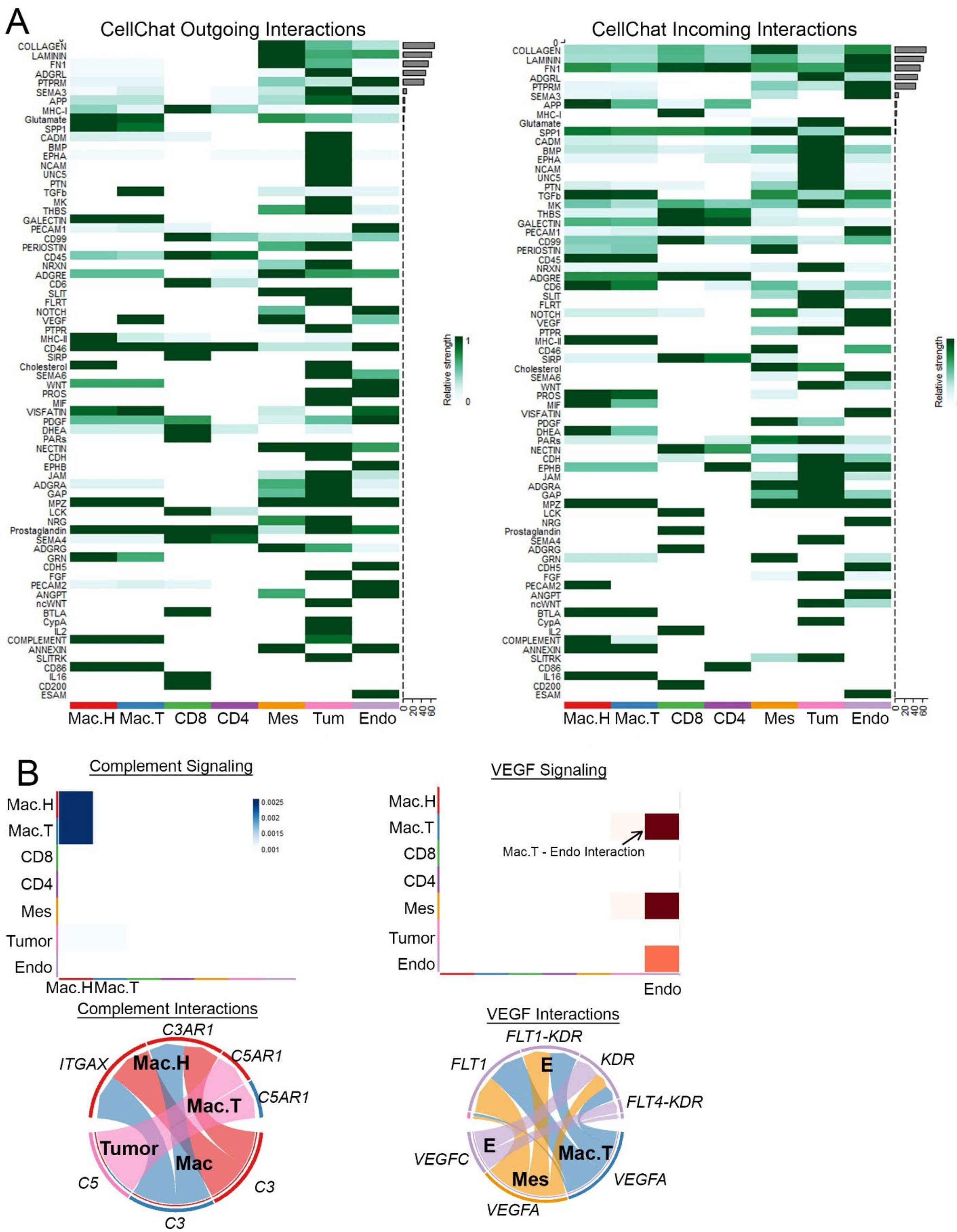

A
CellChat Outgoing Interactions
COLLAGEN
LAMININ
FN1
ADGRL
PTPRM
SEMA3
APP
MHC-I
Glutamate
SPP1
CADM
BMP
EPHA
NCAM
UNC5
PTN
TGFb
MK
THBS
GALECTIN
PECAM1
CD99
PERIOSTIN
CD45
NRXN
ADGRE
CD6
SLIT
FLRT
NOTCH
VEGF
PTPR
MHC-II
CD46
SIRP
Cholesterol
SEMA6
WNT
PROS
MIF
VISFATIN
PDGF
DHEA
PARs
NECTIN
CDH
EPHB
JAM
ADGRA
GAP
MPZ
LCK
NRG
Prostaglandin
SEMA4
ADGRG
GRN
CDH5
FGF
PECAM2
ANGPT
ncWNT
BTLA
CypA
IL2
COMPLEMENT
ANNEXIN
SLITRK
CD86
IL16
CD200
ESAM
Relative strength
1
0
0 20 40 60
Mac.H Mac.T CD8 CD4 Mes Tum Endo
CellChat Incoming Interactions
COLLAGEN
LAMININ
FN1
ADGRL
PTPRM
SEMA3
APP
MHC-I
Glutamate
SPP1
CADM
BMP
EPHA
NCAM
UNC5
PTN
TGFb
MK
THBS
GALECTIN
PECAM1
CD99
PERIOSTIN
CD45
NRXN
ADGRE
CD6
SLIT
FLRT
NOTCH
VEGF
PTPR
MHC-II
CD46
SIRP
Cholesterol
SEMA6
WNT
PROS
MIF
VISFATIN
PDGF
DHEA
PARs
NECTIN
CDH
EPHB
JAM
ADGRA
GAP
MPZ
LCK
NRG
Prostaglandin
SEMA4
ADGRG
GRN
CDH5
FGF
PECAM2
ANGPT
ncWNT
BTLA
CypA
IL2
COMPLEMENT
ANNEXIN
SLITRK
CD86
IL16
CD200
ESAM
Relative strength
1
0
0 20 40 60
Mac.H Mac.T CD8 CD4 Mes Tum Endo
B
Complement Signaling
Mac.H
Mac.T
CD8
CD4
Mes
Tumor
Endo
0.0025
0.002
0.0015
0.001
Mac.H Mac.T
VEGF Signaling
Mac.H
Mac.T
CD8
CD4
Mes
Tumor
Endo
Mac.T - Endo Interaction
Endo
Complement Interactions
C3AR1
ITGAX
Mac.H
C5AR1
C5AR1
Mac.T
Tumor
Mac
C5
C3
C3
VEGF Interactions
FLT1-KDR
FLT1
E
KDR
FLT4-KDR
E
VEGFC
Mac.T
Mes
VEGFA
VEGFA

Figure S9. CellChat analysis of predicted ligand-receptor interactions between macrophages and tumor cells and other cells in the tumor microenvironment in brain metastasis. (A) Heatmap showing all of the predicted outgoing / ligand (left) and incoming / receptor (right) interactions between cell types in the brain metastasis microenvironment (Mac.H = HLA-DR+ macrophages, Mac.T = TGFB+ macrophages, CD8 T cells, CD4 T cells, mesenchymal cells, tumor cells, and endothelial cells. (B) There is an increase in predicted complement signaling between HLA-DR+ macrophages and other macrophages in the microenvironment and an increase in VEGF signaling between TGFβ+ macrophages and endothelial cells in the microenvironment.

Table S2. Cell counts by sample for each UMAP cluster in Figures 1 and 3.

**Sample #**	**Origin**	**Total Cell Count**	Tumor Prolif	Tumor	Mes.	Lymph.	Myeloid	Endo.	Astrocyte
			**Tumor Cell Clusters (Figure 1)**						
07-0649	Lung	8748	2996	5657	54	3	19	19	0
09-0493	Breast	9699	1722	7768	24	26	127	31	1
09-0602	Lung	1924	85	1331	64	30	368	44	2
10-0264	Breast	6283	289	5245	278	18	236	175	42
11-0092	Skin	8505	699	7308	25	209	235	27	2
11-0418	Skin	7836	211	5239	114	1406	800	65	1
11-0430	Breast	8237	1804	6063	223	10	56	66	15
12-0235	Lung	6072	164	5634	34	56	161	9	14
13-0140	Lung	1714	26	1450	25	8	142	38	25
14-0124	Lung	4686	437	3902	38	12	221	27	49
14-0228	Lung	463	31	386	6	6	30	2	2
15-0065	Breast	4204	866	2982	116	7	95	65	73
15-0112	Breast	856	75	764	6	2	5	3	1
15-0143	Lung	2093	508	1410	86	9	40	39	1
15-0215	Skin	7292	568	6239	28	186	231	23	17
16-0065	Lung	1934	768	1132	2	4	17	2	9
16-0144	Lung	1934	47	2329	54	15	22	10	54
17-0250	Lung	3410	68	3137	71	17	35	40	42
18-0126	Breast	7981	471	7148	57	63	195	11	36
18-0137	Breast	4501	593	3019	340	31	235	280	3
19-0016	Lung	3712	570	2547	75	114	342	55	9
19-0145	Breast	6030	812	4761	58	170	193	31	5
19-0324	Lung	5886	1905	3695	38	143	89	12	4
		114000	15715	89146	1816	2545	3894	1074	407

Tumor Prolif. = Tumor Proliferating

Mes. = Mesenchymal

Lymph. = Lymphocytes

Endo. = Endothelial cells

Table S2 (continued)

Sample #	Origin	Total Cell Count	CD8 T Cell	CD4 T Cell	B Cells	Myeloid	Endo	FB	SMC	Astrocyte	Glial Cell	Mix
			Microenvironment Cell Clusters (Figure 3)									
07-0649	Lung	8748	0	0	2	16	17	41	7	12	0	0
09-0493	Breast	9699	16	0	0	62	8	2	5	0	17	120
09-0602	Lung	1924	0	16	0	368	45	4	58	1	0	16
10-0264	Breast	6283	0	6	0	174	107	3	14	10	2	435
11-0092	Skin	8505	0	0	2	0	0	11	4	1	7	1
11-0418	Skin	7836	466	655	120	722	47	75	29	0	0	27
11-0430	Breast	8237	1	1	0	49	53	21	155	12	3	83
12-0235	Lung	6072	1	28	0	168	8	3	25	13	7	29
13-0140	Lung	1714	0	3	1	147	35	1	7	22	2	22
14-0124	Lung	4686	0	7	0	233	28	1	29	51	23	0
14-0228	Lung	463	0	1	0	28	2	1	5	3	6	6
15-0065	Breast	4204	0	4	0	77	63	48	53	98	45	0
15-0112	Breast	856	0	0	0	9	3	0	2	3	0	0
15-0143	Lung	2093	0	3	0	37	38	54	31	12	1	0
15-0215	Skin	7292	57	78	71	194	22	18	9	16	18	22
16-0065	Lung	1934	0	2	0	13	1	0	1	17	85	0
16-0144	Lung	1934	0	0	0	6	3	1	1	88	0	61
17-0250	Lung	3410	0	1	2	40	37	1	58	61	3	7
18-0126	Breast	7981	1	43	7	196	11	4	45	35	108	18
18-0137	Breast	4501	0	3	0	211	228	162	150	0	0	5
19-0016	Lung	3712	0	1	0	466	50	56	14	7	0	1
19-0145	Breast	6030	0	155	3	185	27	30	15	5	9	8
19-0324	Lung	5886	6	106	21	85	12	9	25	22	17	0
		114000	548	1113	229	3486	845	546	742	489	353	861

Endo. = Endothelial cells

FB = Fibroblasts

SMC = Smooth muscle cells

Table S3. Complete Xenium spatial transcriptomic gene panel

Name	Cell	Name	Cell	Name	Cell	Name	Cell
ADH1B	Adipocytes	ANKRD28	B cells	ABCC11	Breast cancer	ADAM9	Breast glandular
ADIPOQ	Adipocytes	APOBEC3A	B cells	APOBEC3B	Breast cancer	AGR3	Breast glandular
ANKRD29	Adipocytes	BANK1	B cells	AR	Breast cancer	ALDH1A3	Breast glandular
CAVIN2	Adipocytes	C2orf42	B cells	CAV1	Breast cancer	AQP3	Breast glandular
CLEC14A	Adipocytes	CCPG1	B cells	CCND1	Breast cancer	BACE2	Breast glandular
EGFL7	Adipocytes	CD19	B cells	CDH1	Breast cancer	C15orf48	Breast glandular
HOXD8	Adipocytes	CD27	B cells	CEACAM6	Breast cancer	C5orf46	Breast glandular
HOXD9	Adipocytes	CD79A	B cells	CENPF	Breast cancer	CCDC6	Breast glandular
KDR	Adipocytes	CD79B	B cells	CXCL12	Breast cancer	CD9	Breast glandular
LPL	Adipocytes	CLECL1	B cells	EGFR	Breast cancer	CDC42EP1	Breast glandular
MMRN2	Adipocytes	DNAAF1	B cells	ERBB2	Breast cancer	CLIC6	Breast glandular
NDUFA4L2	Adipocytes	DUSP5	B cells	ESR1	Breast cancer	CTTN	Breast glandular
NOSTRIN	Adipocytes	ERN1	B cells	FOXA1	Breast cancer	DAPK3	Breast glandular
PDCD1LG2	Adipocytes	FAM107B	B cells	GATA3	Breast cancer	DMKN	Breast glandular
PDE4A	Adipocytes	ITM2C	B cells	KIT	Breast cancer	DNTTIP1	Breast glandular
RAMP2	Adipocytes	LGALSL	B cells	KRT7	Breast cancer	DSC2	Breast glandular
RAPGEF3	Adipocytes	MS4A1	B cells	LDHB	Breast cancer	EIF4EBP1	Breast glandular
SOX18	Adipocytes	MZB1	B cells	LRRC15	Breast cancer	ENAH	Breast glandular
TFAP2A	Adipocytes	PELI1	B cells	MDM2	Breast cancer	FASN	Breast glandular
TIMP4	Adipocytes	RAB30	B cells	MKI67	Breast cancer	FLNB	Breast glandular
UCP1	Adipocytes	SEC11C	B cells	PCLAF	Breast cancer	HMGA1	Breast glandular
		SLAMF1	B cells	PGR	Breast cancer	HOOK2	Breast glandular
		SLAMF7	B cells	PTGDS	Breast cancer	HPX	Breast glandular
		TCL1A	B cells	RUNX1	Breast cancer	JUP	Breast glandular
		TRIB1	B cells	S100A4	Breast cancer	KRT23	Breast glandular
		VOPP1	B cells	SCD	Breast cancer	LYPD3	Breast glandular
		TNFRSF17	Plasma	SFRP1	Breast cancer	MLPH	Breast glandular
				SNAI1	Breast cancer	MYO5B	Breast glandular
				TACSTD2	Breast cancer	OCIAD2	Breast glandular
				TCF4	Breast cancer	OPRPN	Breast glandular
				TOP2A	Breast cancer	PIGR	Breast glandular
				ZEB1	Breast cancer	PTRHD1	Breast glandular
				ZEB2	Breast cancer	QARS	Breast glandular
						S100A14	Breast glandular
						SCGB2A1	Breast glandular
						SDC4	Breast glandular
						SERHL2	Breast glandular
						SERPINA3	Breast glandular
						SH3YL1	Breast glandular
						SLC25A37	Breast glandular
						SLC5A6	Breast glandular
						SMS	Breast glandular
						SQLE	Breast glandular
						SRPK1	Breast glandular
						SVIL	Breast glandular
						TCIM	Breast glandular
						TMEM147	Breast glandular
						TOMM7	Breast glandular
						TPD52	Breast glandular
						TRAPPC3	Breast glandular
						USP53	Breast glandular
						ZNF562	Breast glandular

Table S3 (continued)

Name	Cell	Name	Cell	Name	Cell	Name	Cell
ACTG2	Breast myoepithelial	ADGRE5	Dendritic	AKR1C3	Endothelial	CD247	NK cells
C6orf132	Breast myoepithelial	CD83	Dendritic	ANGPT2	Endothelial	FCER1G	NK cells
DSP	Breast myoepithelial	CLEC9A	Dendritic	AQP1	Endothelial	GNLY	NK cells
DST	Breast myoepithelial	FCER1A	Dendritic	CD93	Endothelial	KLRD1	NK cells
GLIPR1	Breast myoepithelial	IL2RG	Dendritic	ESM1	Endothelial	KLRF1	NK cells
KARS	Breast myoepithelial	IL3RA	Dendritic	PECAM1	Endothelial	NCAM1	NK cells
KLF5	Breast myoepithelial	ITGAX	Dendritic	SOX17	Endothelial	NKG7	NK cells
KRT5	Breast myoepithelial	LILRA4	Dendritic	STC1	Endothelial	PRF1	NK cells
KRT6B	Breast myoepithelial	SPIB	Dendritic	TIFA	Endothelial	CEACAM8	Neutrophils
MYLK	Breast myoepithelial	PTPRC	Immune	VWF	Endothelial	CCL5	T cells
NARS	Breast myoepithelial	AIF1	Macrophages	WARS	Endothelial	CCR7	T cells
OXTR	Breast myoepithelial	APOC1	Macrophages	ANKRD30A	Epithelial	CD274	T cells
PTN	Breast myoepithelial	BASP1	Macrophages	EPCAM	Epithelial	CD3E	T cells
TRAF4	Breast myoepithelial	C1QA	Macrophages	KRT14	Epithelial	CD3G	T cells
TUBB2B	Breast myoepithelial	C1QC	Macrophages	KRT8	Epithelial	CD4	T cells
		CD163	Macrophages	MUC6	Epithelial	CD69	T cells
		CD68	Macrophages	MYBPC1	Epithelial	CD8A	T cells
		CD80	Macrophages	CCDC80	Fibroblasts	CTLA4	T cells
		CD86	Macrophages	DPT	Fibroblasts	CXCR4	T cells
		CX3CR1	Macrophages	FBLN1	Fibroblasts	CYTIP	T cells
		CXCL16	Macrophages	IGF1	Fibroblasts	DUSP2	T cells
		CXCL5	Macrophages	LUM	Fibroblasts	FOXP3	T cells
		FAM49A	Macrophages	MMP1	Fibroblasts	GPR183	T cells
		FGL2	Macrophages	MMP12	Fibroblasts	GZMA	T cells
		GJB2	Macrophages	MMP2	Fibroblasts	GZMK	T cells
		IGSF6	Macrophages	PDGFRA	Fibroblasts	HAVCR2	T cells
		LPXN	Macrophages	PIM1	Fibroblasts	IL2RA	T cells
		LY86	Macrophages	POSTN	Fibroblasts	IL7R	T cells
		LYZ	Macrophages	SFRP4	Fibroblasts	KLRB1	T cells
		MAP3K8	Macrophages	TAC1	Fibroblasts	KLRC1	T cells
		MNDA	Macrophages	TCEAL7	Fibroblasts	LAG3	T cells
		MRC1	Macrophages	ACTA2	Smooth muscle	LTB	T cells
		PLD4	Macrophages	AKR1C1	Smooth muscle	PDCD1	T cells
		PPARG	Macrophages	AVPR1A	Smooth muscle	PRDM1	T cells
		SEC24A	Macrophages	CCL8	Smooth muscle	RHOH	T cells
		SERPINB9	Macrophages	CRISPLD2	Smooth muscle	RORC	T cells
		CPA3	Mast cells	CTH	Smooth muscle	RTKN2	T cells
		CTSG	Mast cells	FOXC2	Smooth muscle	SELL	T cells
		TPSAB1	Mast cells	FSTL3	Smooth muscle	SMAP2	T cells
		CD14	Monocytes	LARS	Smooth muscle	TCF7	T cells
		FCGR3A	Monocytes	MYH11	Smooth muscle	TENT5C	T cells
		S100A8	Monocytes	PDGFRB	Smooth muscle	TIGIT	T cells
		ITGAM	Myeloid cells	PDK4	Smooth muscle	TRAC	T cells
				SSTR2	Smooth muscle	TUBA4A	T cells
				THAP2	Smooth muscle		
